# Biofeedback in the prophylactic treatment of medication overuse headache: a pilot randomized controlled trial

**DOI:** 10.1186/s10194-016-0679-9

**Published:** 2016-09-22

**Authors:** Marialuisa Rausa, Daniela Palomba, Sabina Cevoli, Luana Lazzerini, Elisa Sancisi, Pietro Cortelli, Giulia Pierangeli

**Affiliations:** 1 Department of Biomedical and Neuromotor Sciences DIBINEM, University of Bologna, Bologna, Italy; 2 Service for the Diagnosis and Treatment of Eating Disorders, Service for the Diagnosis and Treatment of Anxiety and Psychosomatic Disorders, Centro Gruber, Bologna, Italy; 3 Department of General Psychology, University of Padova, Padova, Italy; 4 Padiglione G, Bellaria Hospital, IRCCS Institute of Neurological Sciences of Bologna, Via Altura 3, 40139 Bologna, Italy; 5 Neurology, AUSL (Local Health Service) of Ferrara, Ferrara, Italy

**Keywords:** Biofeedback, Coping strategies, Psychological treatment, Chronic migraine, Medication overuse headache, Preventive therapy

## Abstract

**Background:**

Medication overuse headache (MOH) is a major clinical concern and a common health risk. Recent literature stressed the need to manage chronic headache by using integrated biobehavioral approaches. Few studies evaluated how biofeedback can be useful in MOH.

The aim of the study is to evaluate in a randomized, controlled, single-blind trial the effects of biofeedback associated with traditional pharmacological therapy in the prophylactic treatment of MOH.

**Method:**

Twenty-seven subjects were randomized to frontal electromyographic (EMG) biofeedback associated with prophylactic pharmacological therapy (Bfb Group) or to pharmacological treatment alone (Control Group). The primary outcome was to evaluate the number of patients that return episodic after treatment. Secondly we evaluate the effects of frontal EMG BFB on frequency of headache and analgesic intake. Changes in coping strategies and in EMG frontalis tension were also evaluated. ANOVA was performed on all the variables of interest.

**Results:**

Our results indicate that at the end of treatment the number of patients that returned episodic in the Bfb group was significantly higher than in the Control group. Patients in the Bfb group differed from the Control group in headache frequency, amount of drug intake and active coping with pain. These outcomes were confirmed also after 4 months of follow-up. No significant effects were observed in EMG recordings.

**Conclusions:**

Biofeedback added to traditional pharmacological therapy in the treatment of MOH is a promising approach for reducing headache frequency and analgesic intake. Modification of coping cognitions in the Bfb group, as an adjunct mechanism of self-regulation, needs more evaluations to understand the role of biofeedback in changing maladaptive psychophysiological responses.

**Electronic supplementary material:**

The online version of this article (doi:10.1186/s10194-016-0679-9) contains supplementary material, which is available to authorized users.

## Background

Chronic headache comprise individuals with chronic tension-type headache (CTTH) and chronic migraine (CM), both of which may be associated with medication overuse and medication overuse headache (MOH) [1].

In particular, MOH is a major clinical concern and a common health risk [2]. It has a prevalence of about 1–2 % in the general population [3]. The International Classification of Headache Disorders 3rd edition (beta version) suggests a double diagnosis of either CM or CTTH and MOH, since MOH is excluded or confirmed by analgesic withdrawal.

The treatment of MOH is often complex and includes patient education, discontinuation of the offending drug, rescue therapy for withdrawal symptoms, and preventive therapy [4]. In particular, the withdrawal of the overused medication is recognized as the treatment of choice [5–10]. Such a treatment is often compromised by lack of motivation and poor patient’s self-awareness [11].

In the literature [12–14] the basic psychological factors that are key contributors to MOH are described as follows: a belief that acute medication is the only treatment option, the presence of cephalalgiaphobia [12] (or pain panic, i.e. anticipatory fear of pain), intolerance or difficulty dealing with pain, soporophilia (seeking sedation), the need to maintain the usual daily activities, presence of outside pressures and of psychiatric comorbidities.

Chronic headaches should be treated with multidimensional approaches that can support patients not only pharmacologically but also giving them behavioral and cognitive strategies to cope with their pain [15]. One of the non-pharmacological treatments that has shown positive results in treating migraine and tension-type headache is biofeedback [16–20]. Electromyographic biofeedback (EMG BFB) has proved to be effective in reducing pain symptoms associated with both tension-type headache and migraine [17, 19]. Moreover, it has been shown that many forms of headache, especially if chronic, eventually end up in a mixed headache type, and may meet the criteria for TTH [21, 22]. Although there is a large amount of scientific evidence on biobehavioral therapies for headache [23], few studies evaluated how psychological treatments could be integrated with pharmacological prophylaxis in order to favor the reduction of acute medication intake [18, 21]. Only one study [24] evaluated the effects of EMG BFB in a combined treatment of transformed migraine with analgesic overuse. Data showed that patients treated with biofeedback and pharmacological therapy, after analgesic withdrawal, improved in headache frequency and analgesic intake similarly to control group, but have better improvement after 3 years of follow-up..

No data are available about the role that biofeedback treatment could have in reducing medication overuse without a structured drug withdrawal. Moreover, it has not yet been investigated if biofeedback, as an active self-regulation intervention, could also help MOH patients by changing their strategies to cope with headache attacks in comparison with pharmacological treatment.

Indeed, the role of coping strategies in managing chronic pain and headache has been previously investigated [25–29]. In particular, some studies explored the role of pain catastrophizing (as a maladaptive coping strategy) in migraine. Catastophizing is associated with impaired quality of life [26], chronicity of headache and poorer treatment response [27, 28]; whereas few studies analyzed the effects of biofeedback treatment on coping skills [16].

In his meta-analysis on efficacy of BFB on migraine, Nestoriuc found that self-efficacy yielded higher effect sizes than the actual pain related outcome measures of biofeedback and recommended studies to directly investigate whether changes in self-efficacy (and subsequent changes in coping strategies) mediate the treatment effects of BFB. Currently, no data are available on modifications in coping skills after biofeedback training in MOH.

The aims of the present study were to evaluate the effects of EMG BFB associated with traditional pharmacological interventions on patients with MOH without previous withdrawal intervention in a tertiary headache center. The primary outcome was to evaluate the number of patients that return episodic after treatment. Secondly we evaluate the effects of frontal EMG BFB on frequency of headache and analgesic intake. Changes in coping strategies and in EMG frontalis tension were also evaluated.

## Method

### Participants

All consecutive patients attending the Headache Center of IRCCS Institute of Neurological Sciences of Bologna in a range of 2 years (from 2008 to 2010), satisfying inclusion criteria for CM and MOH or CTTH and MOH, and accepting to participate were recruited. Headache and drug overuse were classified according to the International Classification of Headache Disorders 3rd Edition (beta version) [1].

Exclusion criteria were: foreign language as mother tongue, pregnancy, secondary headaches, age < 18, noncompliance. Secondary headaches were ruled out by clinical examination, biochemical tests, and neuroimaging studies, when indicated.

Participants gave written informed consent and the study was carried out in accordance with the Declaration of Helsinki; the study protocol was approved by the Ethic Committee of the Local Health Service of Bologna, Italy (protocol number: 07044).

### Protocol

During the first visit (T0) patients who gave informed consent and satisfied the inclusion criteria received a headache diary, self-administered questionnaires (see Additional file 1: Measures paragraph), and pharmacological therapy prescription. The pharmacological prophylaxis was chosen by the neurologist according to the prophylactic therapy best suited to each patient, considering efficacy and side effects of previous treatments, comorbidity, and patient’s preferences. The neurologist also informed patients about the risks of medication overuse, asking to stop or reduce analgesic intake.

After 1 month (T1) psychophysiological measures were recorded at rest. Headache diary and questionnaires were administered again. At the end of the assessment patients were randomly assigned to the treatment group (Bfb group) or to the control group (Control group). Randomization codes were generated through computer and inserted in numerical sequence into sealed envelopes. Subjects were allocated in 1:1 ratio. The psychologist knew patients’ allocation, while neurologists were blinded to it.

Patients in the Bfb group underwent 9 weekly sessions of EMG biofeedback, whereas patients in the Control group underwent 9 weekly sessions with a psychologist in which they were interviewed about their previous week’s headaches, their mood, and their analgesic intake. In both groups patients were encouraged to stop or reduce analgesic overuse. Neurologists were blinded to which group the patients belonged.

At the end of the treatment (T2) patients were evaluated from the neurologist and from the psychologist (by psychophysiological assessment). Headache diary and questionnaires were re-administered. The same procedure was followed after 4 months from the end of the treatment (T3). At 1 year from the end of the treatment (T4) patients were visited and evaluated by a neurologist.

### Measures

Each time (T0, T1, T2, T3) patients were evaluated by the following measures.

The *Headache Diary* is a monthly diary in which frequency (number of days), intensity (from 1 to 3), and duration (number of hours in a day) of headache attacks were recorded along with the type and the amount of analgesic intake.

PRSS (Pain Related Self Statements Scale) and PRCS (Pain Related Control Scale) [30, 31] are two self-administered questionnaires. The PRSS is an 18-item questionnaire that assesses situation-specific aspects of patients’ cognitive coping strategies for pain. Patients have to choose on a Likert scale (0 to 5) how many times they have thoughts such as “If I stay calm and relax I feel better” or “I cannot tolerate this pain anymore”. PRSS has two subscales: “Catastrophizing” and “Active Coping”. The PRCS is a 15-item questionnaire that measures general attitudes towards pain with statements like “I myself can do something against my pain” and it is divided into 2 subscales: ‘Helplessness’ and ‘Resourcefulness’. Both questionnaires were demonstrated to be valid and sensitive to change, and they are closely related to pain intensity and interference from pain experiences.

At T4 the neurologist evaluated the current headache diagnosis and adherence to pharmacological treatment.

### Physiological measures

The frontalis muscle electromyographic activity as a measure of tension was recorded in baseline condition and during the training. The EMG was recorded and fed back to the subject by means of a Biofeedback Modular System (Modulab series 800, SATEM, Rome, Italy).

### Treatment

The EMG BFB treatment was carried out by a psychologist at Centro Gruber (Bologna), a service for the diagnosis and treatment of eating disorders and of anxiety and psychosomatic disorders. The initial psychophysiological assessment consisted of 2 sessions in which both clinical data and psychophysiological recordings were collected to assess the state of the patient before treatment at baseline and under stress conditions. The 9 weekly sessions of frontalis muscle EMG BFB aimed to reduce muscle tension. The treatment was divided into three phases: a first acquisition phase, in which the feedback was always present (3 sessions), a second maintenance phase, in which trials with and without feedback were alternated (3 sessions), and a third exposure phase, in which patients attempted to use the technique in imagined situations subjectively perceived as stressful (3 sessions). In all phases patients did not were trained in any relaxation technique, they were encouraged to find muscle tension reduction strategies by themselves. The treatment ended with a reassessment session. The procedure of the entire treatment (12 one hour weekly sessions in all)was standardized and was the same for all patients. The psychologist had to fill a checklist of the status of adherence with the protocol.

### Data analysis

The assumptions for the calculation of the sample needed to test the primary end point were: a beta error of 20 %, an alpha error of 5, 50 % of responders in the experimental group and 10 % of responders in the placebo group. Given these assumptions, at least 24 patients per group had to be recruited.

Descriptive statistics (means ± SD) were conducted on the sample features. The sample was randomized into two groups: Biofeedback group (Bfb Group) and Control group. The normality of parameters distribution was checked using Skewedness-Kurtosis. Chi-squared, Student’s *T*-test and ANOVA repeated measures were performed to compare data between groups at T1, T2 and T3. Bonferroni correction for multiple comparisons was used. When appropriate, post-hoc analyses were carried out. Data were analyzed using the statistical software SPSS 19.0 (Statistical Package for Social Science). Significance level was set at two-tailed *p* < 0.05.

## Results

Forty-seven of 72 patients with MOH consecutively referred to the Headache Center responded to the inclusion criteria and accepted to participate. Three patients were excluded because at T1 they did not fulfill the MOH diagnosis anymore, 10 participants dropped out between T1 and T2 (6 in the Bfb group, 4 in the Control group).

**Fig. 1:**
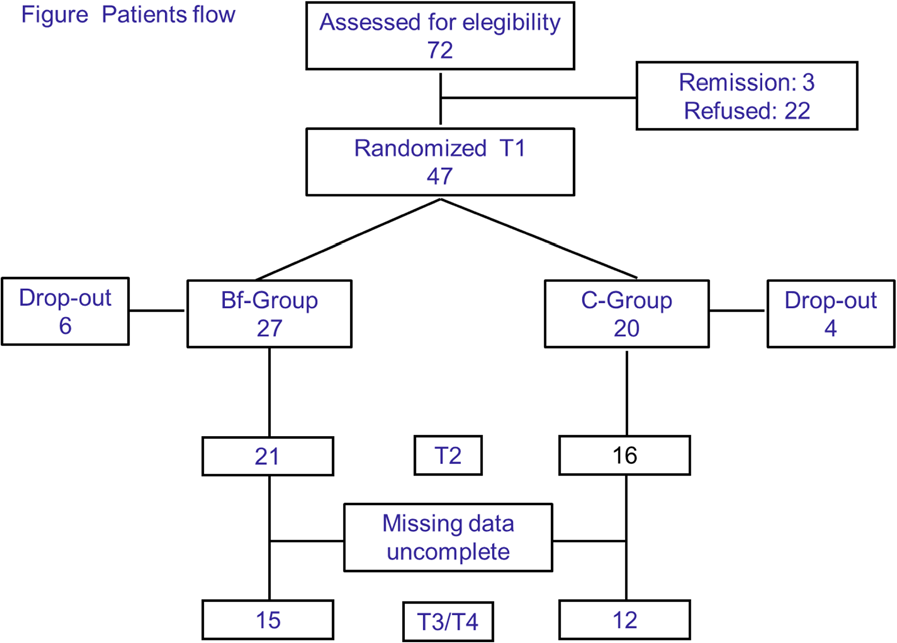
Patients Flow

Analyses were conducted only in the 27 participants who provided headache diary data for all measurement periods and completed the study: 15 belonged to the Bfb group, 12 belonged to Control group (Fig. 1).

Patients who did not complete the study did not differ from other participants in: age (*t* = −.500, *p* = .620), sex (*χ*^2^ = 0.688, *p* = 0.41) educational level (*χ*^2^ = 0.719, *p* = 0.69) and type of headache at onset (*χ*^2^ = 1.340, *p* = 0.51), but they differed in age of chronification (*t* = 3.924, *p* < 0.001): participants who dropped out of the study had suffered from MOH for a longer period of time.

According to the original ICHD 3rd edition (beta version), 24 of 27 subjects had a diagnosis of CM and MOH and 3 of CTTH and MOH.

Table 1 shows patients’ demographic and headache characteristics. Participants of the Bfb group and Control group did not differ in age (*t* = −1.060, *p* = 0.30), sex (*χ*^2^ = 0.059, *p* = 0.81), educational level (*χ*^2^ = 0.617, *p* = 0.73), type of headache at onset (*χ*^2^ = 1.739, *p* = 0.42), age of chronification (*t* = −1.025, *p* = 0.31). Drugs used for preventive therapy were: antiepileptics (14 patients: 9 in Bfb group and 5 in Control group), beta-blockers (5 patients: 2 in Bfb group and 3 in Control group), antidepressants (6 patients: 3 in Bfb group and 3 in Control group), Ca-antagonists (2 patients in Control group), pizotifen (1 patient in Bfb group).

**Table 1:** Patients’ demographic and headache characteristics

	Biofeedback Group	Control Group	Total
Gender			
Male	2	2	4
Female	13	10	23
Age (mean ± sd)	40.13 ± 12.41	45.08 ± 11.60	42.33 ± 12.09
Educational Level			
Primary/Secondary	2	3	5
High School	4	3	7
Graduate	9	6	15
Headache at onset			
MWOA	11	10	21
MWA	2	0	2
TTH	2	2	4
Age of chronification (mean ± sd)	28.53 ± 9.96	32.25 ± 8.85	30.18 ± 9.49
Overused drugs			
Triptans	7	5	12
NSAIDs/P	2	0	2
Combination-analgesics	1	2	3
Multiple drug classes	5	5	10
Drug prophylaxis			
Monotherapy	9	7	16
Polytherapy	4	5	9

*Abbreviations*: *MWOA* migraine without aura, *MWA* migraine with aura, *TTH* tension type headache

At the end of the treatment (T2) patients that returned to episodic headache were respectively 10 (67 %) in the Bfb group and 2 (17 %) in the Control group (*χ*^2^ = 6.750, *p* = 0.009); after 4 months (T3), 12 (80 %) in the Bfb group and 3 (25 %) in the Control group (*χ*^2^ = 8.168, *p* = 0.004). After 1 year (T4), 7 patients (47 %) in the Bfb group and 2 (17 %) in the Control group (*χ*^2^ = 2.700, *p* = 0.10) remained episodic (Table 2).

**Table 2:** Frequencies and percentages of responders (patients that returned episodic) at the end of treatment (T2), after 4 months (T3) and at 1 year (T4)

Responders	Biofeedback group	Control group
T2		
*n* (%)	10 (67 %)	2 (17 %)
T3		
*n* (%)	12 (80 %)	3 (25 %)
T4		
*n* (%)	7 (47 %)	2 (17 %)

Intention-to treat-analysis was performed on number of responders in the two groups. Including patients that did not complete the study as non responders, the percentages of patients that return episodic at the end of the treatment (T2) were respectively the 37,5 % in the Bfb group, 10 % in the Control Group (*χ*^2^ = 4.417, *p* = 0.036), at T3 44,44 % in the Bfb group and 15 % in the Control Group (*χ*^2^ = 4.584, *p* = 0.032), at T4 25,92 % in the Bfb Group and 10 % in the Control Group (*χ*^2^ = 1,88, *p* = 0.170).

### Diary variables

Analyses were performed on the two groups (15 patients of the Bfb group, 12 patients of the Control group) (Table 3).

**Table 3:** ANOVA results

Clinical index	Biofeedback group			Control group			Group effect	Time effect	Interaction group X time effect
	T1	T2	T3	T1	T2	T3			
	M(CI95%)	M(CI95%)	M(CI95%)	M(CI95%)	M(CI95%)	M(CI95%)			
Frequency (days/month)	19.93 (17.49; 22.37)	13.93 (10.52; 17.34)	13.00 (9.63; 16.37)	22.08 (19.36; 24.81)	24.25 (20.43;28.06)	22.33 (18.56; 26.10)	**F[1,25] = 14.11;	*F[1,25] = 5.44;	**F[1,25] = 9.62;
							*p* = 0.001	*p* = 0.014	*p* = 0.001
Analgesic intake (number tot/month)	21.27 (13.68; 28.85)	11.93 (4.31; 19.53)	12.40 (2.83; 21.97)	26,62 (18.14; 35.11)	27.04 (18.54; 35.54)	21.17 (16.47; 37.86)	F[1,25] = 4.192;	*F[1,25] = 4.52;	*F[1,25] = 5.561
							*p* = 0.051	*p* = 0.025	*p* = 0.012

*Abbreviations*: *M* mean, *CI 95 %* confidence interval 95 %

﻿*p<0.05 **p<0.01

ANOVA repeated measures on attack frequency (number of attacks per month) yielded main effects for time (*p* = 0.014) and group (*p* = 0.001) and a Group X Time interaction effect (*p* = 0.001).

Post-hoc Fisher’s LSD comparisons showed a significant reduction in frequency from T1 to T2 (*p* = 0.002) and from T1 to T3 (*p* < 0.001) only in the Bfb group, no differences in frequency were found in the Control group from T1 to T2 and from T1 to T3. No differences were found between the groups at T1.

Data about the intensity and duration of headache attacks were not analyzed because they were indicated only in 44 % of the diaries filled in by the patients.

Results about analgesic intake showed a main effect for time (*p* < 0.025) and a Group X Time interaction (*p* <0.012). Group effect was close to the significance level, even if it did not reach it (*p* = 0.051).

Post-hoc Fisher’s LSD comparisons showed a significant reduction in analgesic intake from T1 to T2 (*p* = 0.001) and from T1 to T3 (*p* = 0.009) only in the Bfb group, no differences in frequency were found in the Control group from T1 to T2 and from T1 to T3. No differences were found between the groups at T1.

### Psychological measures

PRSS and PRCS results were analyzed in 25 patients (14 in Bfb group, 11 in Control group) because their questionnaires were invalid. In PRSS questionnaire ANOVA repeated measures was performed on the two scales: Catastrophizing and Active Coping. In Catastrophizing, analysis showed a main effect for time (F_[1, 23]_ = 5.762; *p* = 0.006) and group (F_[1, 23]_ = 10.98; *p* = 0.003), but not for the interaction Group x Time (F_[1, 23]_ = .312; *p* = 0.73). On post-hoc comparisons, a significant difference emerged between the groups both in pre (*p* = 0.018) and post-training (*p* = 0.006), at T3 the score decreased in both groups, but only in the Bfb group the decrease was significant (*p* = 0.019).

In the scale Active Coping a time effect (F_[1, 23]_ = 3.984; *p* = 0.044) and a Group X Time interaction (F_[1,23]_ = 4.499; *p* = 0.032) were detected. Post-hoc Fisher’s LSD comparisons showed a significant increase of the scores from T1 to T2 (*p* = 0.002) and from T1 to T3 (*p* = 0.047) only in the Bfb group, no differences in Active Coping were found in the Control group from T1 to T2 (*p* = 1.00) and from T1 to T3 (*p* = 1.00). No differences were found between the groups at T1 (*p* = 0.939).

In PRCS no main interaction effects were detected, only in the subscale Helplessness a group effect was found (F_[1,23]_ = 8.772; *p* = 0.007). On post-hoc comparisons, the Bfb group had a lower score than the Control group at T2 (*p* = 0.036) and at T3 (*p* = 0.004). Means and Confidence intervals are available in the Additional files 1, 2 and 3.

### EMG data

ANOVA repeated measures performed on frontalis EMG in baseline condition at pre- (T1) and post-treatment (T3) did not yield significant results (*p* = 0.52). No differences were found overall between groups and between the groups at T1 and at T3. No differences were found overall between groups and between the groups at T1 (Bfb Group = 2,47 ± 1,10 μV; Control Group = 2,88 ± 1,25 μV) and at T3 (Bfb Group = 2,28 ± 0,60 μV; Control Group = 2,73 ± 1,17 μV).

## Discussion

Our results indicate that at the end of treatment the Bfb group had reduced the headache frequency and the amount of drug intake and showed better active coping with pain, compared with the Control group. These outcomes were confirmed also after 4 months of follow-up.

These results are in line with previous studies, showing that a combined treatment (biofeedback plus pharmacological therapy) for MOH is more effective than pharmacological therapy alone in reducing pain symptoms even in the long run [24]. The combined treatment (biofeedback and pharmacological therapy) was compared with pharmacological treatment after 10 days of drug inpatient withdrawal. Headache frequency and analgesic intake improved even after 3 years of follow-up. Further findings have been obtained from the present study.

First, patients could modify analgesic intake with biofeedback independently of an analgesic-overuse structured withdrawal.

In recent literature the role of drug detoxification has been debated [32–35]. Saper and Lake created a classification system that can be useful in triaging these patients. Type 1 MOH refers to simpler cases of patients who do not have behavioral impairments and do not overuse opioids and barbiturates. Type 2 MOH patients are complex and suffer from behavioral conditions or chronically use opioids or barbiturates [36]. In our study we did not differentiate between simple or complex cases, in future research it could be useful to evaluate such distinction in order to understand which type of patients could benefit from biofeedback treatment. At 1 year of follow-up only 46 % of patients did not return chronic, this result is in line with studies that indicated a high level of relapse in MOH [37, 38] but it would have been better supported if the distinction described above had been used. Moreover, the duration of chronification is one of the characteristics that differed in patients that dropped out of the study. In future research this factor should be taken into account in order to understand if this type of patients need motivational intervention before starting biofeedback treatment or if they could not benefit from this treatment at all.

Second, in our experimental design, patients of the Control and Bfb group were followed up weekly and they were encouraged to stop or reduce analgesic overuse, but our control patients did not benefit from simple advice and support [34], indicating that in order to help patients in reducing drug intake it is necessary to give them different strategies to cope with pain, and biofeedback could help them achieve new self-regulatory strategies.

Third, patients in the Bfb group changed coping cognitions after treatment, they reported using more active coping cognitions than the Control group. Active coping cognitions included thoughts like “If I stay calm and relax, things will be better” or “I can do something about my pain” “I’ll manage” “or “I will cope with it”. Active coping is opposite to pain catastrophizing, which generally refers to exaggerated negative cognitive and affective reactions to an expected or actual pain experience [39] and it is characterized by magnification of the potential negative aspects of pain, inability to disengage from thoughts about pain, and a feeling of helplessness in coping with pain [40].

It has been stressed that pain catastrophizing may also worsen the experience of pain through physiological and neural pathways by enhancing it via differential patterns of brain activation [41] and by modulating the analgesic effects of medications affecting the endogenous opioid system [42, 43]. Our data allowed us to argue that the acquisition of self-regulation strategies could probably help patients to promote active behaviors and to activate different problem solving strategies. The meta-analysis of Nestoriuc supports this hypothesis, showing that frequency of migraine attacks and perceived self-efficacy had the strongest improvements after the treatment [16].

Nonetheless, modifications of coping cognitions in the biofeedback treatment of MOH needs more evaluations to understand the role of biofeedback in changing coping skills.

Fourth, these results were independent of psychophysiological modification, in fact no differences were found in EMG frontalis muscle level at rest after treatment. It could be hypothesized that if sensors had been placed differently (e.g., on the trapezius muscle or on the site most associated with pain), that would have led to more specific EMG results. Moreover, given the effects of BFB training on patients’ coping strategies it could be speculated that BFB acted as a general arousal control strategy [44]. Therefore, in future studies, EMG feedback should be compared with skin conductance or thermal feedback, which more closely reflect the subject’s arousal.

The most important limit of this study is the number of participants that concluded it, as the sample size determined by the calculation of the sample had not been reached. It was difficult to recruit patients and, in particular, to obtain all data for two main reasons: the protocol’s length and the poor compliance that characterizes this kind of patients [11]. For this reason we considered it a pilot study.

Another limit was that at 1 year of follow-up we could not analyze frequency and intensity of headache attacks due to missing data. Also psychological measures could not be collected. Moreover, at 1 year the number of patients that had returned to episodic headache was reduced in the Bfb group and the difference with the control group did not remain significant.

After 1 year patients generally referred that they were not practicing self-regulatory strategies anymore. In future biofeedback protocols it could be useful to plan “recall sessions” at 4, 6, and 9 months and at 1 year from the end of treatment in order to prevent both relapse and missing data.

The sample of the present study included also patients with CTTH, however it was not possible to evaluate differences in the response to treatment according to diagnosis differences, due to the small number of the CTTH sample.

## Conclusions

The results of our study encourage the use of biofeedback in combination with pharmacotherapy in order to stop or reduce analgesic drug overuse. Biofeedback added to traditional pharmacological therapy in the treatment of MOH is a promising approach for reducing the frequency of analgesic intake. Our study also stressed how complex the study of MOH patients is, suggesting that a multicenter randomized control trial could be useful to establish biofeedback efficacy in this kind of patients.

## Additional files

Additional file 1: Questionnaires results. (DOCX 63 kb) Additional file 2: Statistical analysis on headache variables. (RTF 1850 kb) Additional file 3: Statistical analysis on questionnaires variables. (PDF 281 kb)

## Abbreviations

CM: Chronic migraine
CTTH: Chronic tension-type headache
EMG BFB: Electromyographic biofeedback
MOH: Medication overuse headache
