# Supplementary material for: Biofeedback in the prophylactic treatment of medication overuse headache: a pilot randomized controlled trial
Source: J Headache Pain. 2016 Sep 22;17(1):87. doi: 10.1186/s10194-016-0679-9 (PMC5031562; doi:10.1186/s10194-016-0679-9)
Supplement: Additional file 1: — Questionnaires results. (DOCX 63 kb) [file 10194_2016_679_MOESM1_ESM.docx]

| **Questionnaire results** | **Biofeedback Group** | | | **Control Group** | | | **Group Effect** | **Time Effect** | **Interaction**  **Group X Time**  **Effect** |
| --- | --- | --- | --- | --- | --- | --- | --- | --- | --- |
|  | **T1** | **T2** | **T3** | **T1** | **T2** | **T3** |  | | |
|  | **M(CI95%)** | **M(CI95%)** | **M(CI95%)** | **M(CI95%)** | **M(CI95%)** | **M(CI95%)** |  |  |  |
| **PRSS**  **Catastrophizing** | 2.76  (2.38;3.14) | 2.22  (1.68;2.66) | 2.04  (1.48;2.59) | 3.46  (3.04;3.89) | 3.19  (2.60;3.69) | 2.97  (2.35;3.59) | ***F[1,23]=10.98;**  **p=0.003** | ***F[1,23]=5.762;**  **p=0.006** | F[1,23]=.312;  p=0.733 |
| **PRSS**  **Active Coping** | 2.63  (2.12;3.14) | 3.32  (2.94;3.71) | 3.25  (2.77;3.74) | 2.66  (2.08;3.23) | 2.51  (2.07;2.94) | 2.81  (2.26;3.36) | F[1,23]=1.948;  p=0.176 | ***F[1,23]=3.984;**  **p=0.044** | ***F[1,23]=4.499;**  **p=0.032** |
| **PRCS Resourcefulness** | 2.52  (2.09;2.95) | 2.69  (2.28;3.11) | 2.72  (2.31;3.12) | 2.58  (2.10;3.06) | 2.86  (2.40;3.33) | 2.73  (2.27;3.19) | F[1,23]=.099;  p=0.755 | F[1,23]=1.519;  p=0.230 | F[1,23]=.182;  p=0.834 |
| **PRCS Helplessness** | 2.35  (1.89;2.83) | 1.93  (1.41;2.44) | 1.72  (1.32;2.11) | 2.93  (2.38;3.49) | 2.78  (2.18;3.38) | 2.68  (2.21;3.14) | ***F[1,23]=8.772;**  **p=0.007** | F[1,23]=2.879;  p=0.066 | F[1,23]=.531;  p=0.591 |
