# Supplementary material for: Biofeedback in the prophylactic treatment of medication overuse headache: a pilot randomized controlled trial
Source: J Headache Pain. 2016 Sep 22;17(1):87. doi: 10.1186/s10194-016-0679-9 (PMC5031562; doi:10.1186/s10194-016-0679-9)
Supplement: Additional file 2: — Statistical analysis on headache variables. (RTF 1850 kb) [file 10194_2016_679_MOESM2_ESM.rtf]

Modello lineare generalizzato

	Note

Output creato	20-JUN-2014 12:23:55	
Commenti	 	
Input	Dati	H:\ CARTELLE PERSONALI\Rausa Marialuisa\Articolo MIO\analisi statistiche\analisi luana\database completato maggio 2014.sav	
 	File di dati attivo	FileDati1	
 	Filtro	<nessuno>	
 	Peso	<nessuno>	
 	Distingui	<nessuno>	
 	N. di righe nel file dati di lavoro	31	
Gestione valori mancanti	Definizione di valore mancante	I valori mancanti definiti dall'utente vengono considerati mancanti.	
 	Casi utilizzati	Le statistiche sono basate su tutti i casi con dati validi di tutte le variabili del modello.	
Sintassi	GLM f_T1 f_T3 f_T4 BY gruppi
  /WSFACTOR=tempo 3 Polynomial
  /MEASURE=frequenza
  /METHOD=SSTYPE(3)
  /EMMEANS=TABLES(gruppi) COMPARE ADJ(BONFERRONI)
  /EMMEANS=TABLES(tempo) COMPARE ADJ(BONFERRONI)
  /EMMEANS=TABLES(gruppi*tempo) COMPARE(gruppi) ADJ(BONFERRONI)
 /EMMEANS=TABLES(gruppi*tempo) COMPARE(tempo) ADJ(BONFERRONI) /PRINT=DESCRIPTIVE
  /CRITERIA=ALPHA(.05)
  /WSDESIGN=tempo
  /DESIGN=gruppi.
	
Risorse	Tempo trascorso	0:00:00,03	


[FileDati1] H:\ CARTELLE PERSONALI\Rausa Marialuisa\Articolo MIO\analisi statistiche\analisi luana\database completato maggio 2014.sav

	Fattori entro soggetticc

Misura: frequenza 
tempo	Variabile dipendente	
1	f_t1	
2	f_t3	
3	f_t4	


	Fattori tra soggetti

 	N	
gruppi	1,00	15	
 	2,00	12	


	Statistiche descrittive

 	gruppi	Media	Deviazione std.	N	
Frequenza	1,00	19,9333	4,35015	15	
 	2,00	22,0833	4,87029	12	
 	Totale	20,8889	4,62712	27	
Frequenza	1,00	13,9333	5,68792	15	
 	2,00	24,2500	7,23784	12	
 	Totale	18,5185	8,17769	27	
Frequenza	1,00	13,0000	5,15475	15	
 	2,00	22,3333	7,58387	12	
 	Totale	17,1481	7,80879	27	


	Test multivariati(b)

Effetto	 	Valore	F	Ipotesi df	Gradi di libertà dell'errore	Sig.	
tempo	Traccia di Pillai	,283	4,737(a)	2,000	24,000	,018	
 	Lambda di Wilks	,717	4,737(a)	2,000	24,000	,018	
 	Traccia di Hotelling	,395	4,737(a)	2,000	24,000	,018	
 	Radice di Roy	,395	4,737(a)	2,000	24,000	,018	
tempo * gruppi	Traccia di Pillai	,341	6,196(a)	2,000	24,000	,007	
 	Lambda di Wilks	,659	6,196(a)	2,000	24,000	,007	
 	Traccia di Hotelling	,516	6,196(a)	2,000	24,000	,007	
 	Radice di Roy	,516	6,196(a)	2,000	24,000	,007	
a  Statistica esatta
b  Disegno: Intercept+gruppi 
 Disegno entro soggetti: tempo


	Test di sfericità di Mauchly(b)

Misura: frequenza 
Effetto entro soggetti	W di Mauchly	Approssimazione chi-quadrato	df	Sig.	Epsilon(a)	
 	 	 	 	 	Greenhouse-Geisser	Huynh-Feldt	Limite inferiore	
tempo	,694	8,783	2	,012	,765	,838	,500	
Verifica l'ipotesi nulla per la quale la matrice di covarianza dell'errore della variabile dipendente trasformata ortonormalizzata è proporzionale a una matrice identità.
a  È possibile utilizzarlo per regolare i gradi di libertà per i test di significatività mediati. I test corretti vengono visualizzati nella tabella dei test sugli effetti entro soggetti.
b  Disegno: Intercept+gruppi 
 Disegno entro soggetti: tempo


	Test degli effetti entro soggetti

Misura: frequenza 
Sorgente	 	Somma dei quadrati Tipo III	df	Media dei quadrati	F	Sig.	
tempo	Assumendo la sfericità	149,964	2	74,982	5,442	,007	
 	Greenhouse-Geisser	149,964	1,531	97,963	5,442	,014	
 	Huynh-Feldt	149,964	1,676	89,482	5,442	,011	
 	Limite inferiore	149,964	1,000	149,964	5,442	,028	
tempo * gruppi	Assumendo la sfericità	265,026	2	132,513	9,618	,000	
 	Greenhouse-Geisser	265,026	1,531	173,126	9,618	,001	
 	Huynh-Feldt	265,026	1,676	158,138	9,618	,001	
 	Limite inferiore	265,026	1,000	265,026	9,618	,005	
Errore(tempo)	Assumendo la sfericità	688,900	50	13,778	 	 	
 	Greenhouse-Geisser	688,900	38,271	18,001	 	 	
 	Huynh-Feldt	688,900	41,898	16,442	 	 	
 	Limite inferiore	688,900	25,000	27,556	 	 	


	Test dei contrasti entro soggetti

Misura: frequenza 
Sorgente	tempo	Somma dei quadrati Tipo III	df	Media dei quadrati	F	Sig.	
tempo	Lineare	148,890	1	148,890	8,335	,008	
 	Quadratico	1,074	1	1,074	,111	,742	
tempo * gruppi	Lineare	172,001	1	172,001	9,629	,005	
 	Quadratico	93,025	1	93,025	9,598	,005	
Errore(tempo)	Lineare	446,592	25	17,864	 	 	
 	Quadratico	242,308	25	9,692	 	 	


	Test degli effetti fra soggetti

Misura: frequenza 
Variabile trasformata: Media 
Sorgente	Somma dei quadrati Tipo III	df	Media dei quadrati	F	Sig.	
Intercetta	29662,114	1	29662,114	396,383	,000	
gruppi	1056,089	1	1056,089	14,113	,001	
Errore	1870,800	25	74,832	 	 	


Medie marginali attese

1. gruppi

	Stime

Misura: frequenza 
gruppi	Media	Errore std.	Intervallo di confidenza 95%	
 	 	 	Limite inferiore	Limite superiore	
1,00	15,622	1,290	12,966	18,278	
2,00	22,889	1,442	19,920	25,858	


	Confronti a coppie

Misura: frequenza 
(I) gruppi	(J) gruppi	Differenza fra medie (I-J)	Errore std.	Sig.(a)	Intervallo di confidenza per la differenza al 95%(a)	
 	 	 	 	 	Limite inferiore	Limite superiore	
1,00	2,00	-7,267(*)	1,934	,001	-11,250	-3,283	
2,00	1,00	7,267(*)	1,934	,001	3,283	11,250	
Basato sulle medie marginali stimate
*  La differenza fra medie è significativa al livello ,05
a  Correzione per confronti multipli: Bonferroni.


	Test univariati

Misura: frequenza 
 	Somma dei quadrati	df	Media dei quadrati	F	Sig.	
Contrasto	352,030	1	352,030	14,113	,001	
Errore	623,600	25	24,944	 	 	
Ciascun F verifica gli effetti semplici di gruppi all'interno di ogni combinazione di livelli degli altri effetti illustrati. Questi test sono basati sui confronti a coppie indipendenti e lineari tra le medie marginali stimate.


2. tempo

	Stime

Misura: frequenza 
tempo	Media	Errore std.	Intervallo di confidenza 95%	
 	 	 	Limite inferiore	Limite superiore	
1	21,008	,888	19,179	22,837	
2	19,092	1,242	16,533	21,651	
3	17,667	1,228	15,138	20,195	


	Confronti a coppie

Misura: frequenza 
(I) tempo	(J) tempo	Differenza fra medie (I-J)	Errore std.	Sig.(a)	Intervallo di confidenza per la differenza al 95%(a)	
 	 	 	 	 	Limite inferiore	Limite superiore	
1	2	1,917	1,140	,315	-1,007	4,841	
 	3	3,342(*)	1,157	,024	,372	6,312	
2	1	-1,917	1,140	,315	-4,841	1,007	
 	3	1,425	,680	,139	-,319	3,169	
3	1	-3,342(*)	1,157	,024	-6,312	-,372	
 	2	-1,425	,680	,139	-3,169	,319	
Basato sulle medie marginali stimate
*  La differenza fra medie è significativa al livello ,05
a  Correzione per confronti multipli: Bonferroni.


	Test multivariati

 	Valore	F	Ipotesi df	Gradi di libertà dell'errore	Sig.	
Traccia di Pillai	,283	4,737(a)	2,000	24,000	,018	
Lambda di Wilks	,717	4,737(a)	2,000	24,000	,018	
Traccia di Hotelling	,395	4,737(a)	2,000	24,000	,018	
Radice di Roy	,395	4,737(a)	2,000	24,000	,018	
Ciascun F verifica l'effetto multivariato di tempo. Questi test sono basati sui confronti a coppie indipendenti e lineari tra le medie marginali stimate.
a  Statistica esatta


3. gruppi * tempo

	Stime

Misura: frequenza 
gruppi	tempo	Media	Errore std.	Intervallo di confidenza 95%	
 	 	 	 	Limite inferiore	Limite superiore	
1,00	1	19,933	1,184	17,494	22,372	
 	2	13,933	1,657	10,521	17,345	
 	3	13,000	1,637	9,629	16,371	
2,00	1	22,083	1,324	19,357	24,810	
 	2	24,250	1,852	20,435	28,065	
 	3	22,333	1,830	18,564	26,102	


	Confronti a coppie

Misura: frequenza 
tempo	(I) gruppi	(J) gruppi	Differenza fra medie (I-J)	Errore std.	Sig.(a)	Intervallo di confidenza per la differenza al 95%(a)	
 	 	 	 	 	 	Limite inferiore	Limite superiore	
1	1,00	2,00	-2,150	1,776	,237	-5,808	1,508	
 	2,00	1,00	2,150	1,776	,237	-1,508	5,808	
2	1,00	2,00	-10,317(*)	2,485	,000	-15,435	-5,199	
 	2,00	1,00	10,317(*)	2,485	,000	5,199	15,435	
3	1,00	2,00	-9,333(*)	2,455	,001	-14,390	-4,277	
 	2,00	1,00	9,333(*)	2,455	,001	4,277	14,390	
Basato sulle medie marginali stimate
*  La differenza fra medie è significativa al livello ,05
a  Correzione per confronti multipli: Bonferroni.


	Test univariati

Misura: frequenza 
tempo	 	Somma dei quadrati	df	Media dei quadrati	F	Sig.	
1	Contrasto	30,817	1	30,817	1,465	,237	
 	Errore	525,850	25	21,034	 	 	
2	Contrasto	709,557	1	709,557	17,236	,000	
 	Errore	1029,183	25	41,167	 	 	
3	Contrasto	580,741	1	580,741	14,451	,001	
 	Errore	1004,667	25	40,187	 	 	
F verifica l'effetto di gruppi. Questo test è basato sui confronti a coppie indipendenti e lineari tra le medie marginali stimate.


4. gruppi * tempo

	Stime

Misura: frequenza 
gruppi	tempo	Media	Errore std.	Intervallo di confidenza 95%	
 	 	 	 	Limite inferiore	Limite superiore	
1,00	1	19,933	1,184	17,494	22,372	
 	2	13,933	1,657	10,521	17,345	
 	3	13,000	1,637	9,629	16,371	
2,00	1	22,083	1,324	19,357	24,810	
 	2	24,250	1,852	20,435	28,065	
 	3	22,333	1,830	18,564	26,102	


	Confronti a coppie

Misura: frequenza 
gruppi	(I) tempo	(J) tempo	Differenza fra medie (I-J)	Errore std.	Sig.(a)	Intervallo di confidenza per la differenza al 95%(a)	
 	 	 	 	 	 	Limite inferiore	Limite superiore	
1,00	1	2	6,000(*)	1,519	,002	2,101	9,899	
 	 	3	6,933(*)	1,543	,000	2,973	10,893	
 	2	1	-6,000(*)	1,519	,002	-9,899	-2,101	
 	 	3	,933	,906	,938	-1,392	3,258	
 	3	1	-6,933(*)	1,543	,000	-10,893	-2,973	
 	 	2	-,933	,906	,938	-3,258	1,392	
2,00	1	2	-2,167	1,699	,642	-6,525	2,192	
 	 	3	-,250	1,725	1,000	-4,678	4,178	
 	2	1	2,167	1,699	,642	-2,192	6,525	
 	 	3	1,917	1,013	,210	-,683	4,516	
 	3	1	,250	1,725	1,000	-4,178	4,678	
 	 	2	-1,917	1,013	,210	-4,516	,683	
Basato sulle medie marginali stimate
*  La differenza fra medie è significativa al livello ,05
a  Correzione per confronti multipli: Bonferroni.


	Test multivariati

gruppi	 	Valore	F	Ipotesi df	Gradi di libertà dell'errore	Sig.	
1,00	Traccia di Pillai	,449	9,776(a)	2,000	24,000	,001	
 	Lambda di Wilks	,551	9,776(a)	2,000	24,000	,001	
 	Traccia di Hotelling	,815	9,776(a)	2,000	24,000	,001	
 	Radice di Roy	,815	9,776(a)	2,000	24,000	,001	
2,00	Traccia di Pillai	,144	2,019(a)	2,000	24,000	,155	
 	Lambda di Wilks	,856	2,019(a)	2,000	24,000	,155	
 	Traccia di Hotelling	,168	2,019(a)	2,000	24,000	,155	
 	Radice di Roy	,168	2,019(a)	2,000	24,000	,155	
Ciascun F verifica gli effetti multivariati semplici di tempo all'interno di ogni combinazione di livelli degli effetti illustrati. Questi test sono basati sui confronti a coppie indipendenti e lineari tra le medie marginali stimate.
a  Statistica esatta


Modello lineare generalizzato

	Note

Output creato	20-JUN-2014 12:24:50	
Commenti	 	
Input	Dati	H:\ CARTELLE PERSONALI\Rausa Marialuisa\Articolo MIO\analisi statistiche\analisi luana\database completato maggio 2014.sav	
 	File di dati attivo	FileDati1	
 	Filtro	<nessuno>	
 	Peso	<nessuno>	
 	Distingui	<nessuno>	
 	N. di righe nel file dati di lavoro	31	
Gestione valori mancanti	Definizione di valore mancante	I valori mancanti definiti dall'utente vengono considerati mancanti.	
 	Casi utilizzati	Le statistiche sono basate su tutti i casi con dati validi di tutte le variabili del modello.	
Sintassi	GLM i_T1 i_T3 i_T4 BY gruppi
  /WSFACTOR=tempo 3 Polynomial
  /MEASURE=intensità
  /METHOD=SSTYPE(3)
  /EMMEANS=TABLES(gruppi) COMPARE ADJ(BONFERRONI)
  /EMMEANS=TABLES(tempo) COMPARE ADJ(BONFERRONI)
  /EMMEANS=TABLES(gruppi*tempo) COMPARE(gruppi) ADJ(BONFERRONI)
 /EMMEANS=TABLES(gruppi*tempo) COMPARE(tempo) ADJ(BONFERRONI) /PRINT=DESCRIPTIVE
  /CRITERIA=ALPHA(.05)
  /WSDESIGN=tempo
  /DESIGN=gruppi.
	
Risorse	Tempo trascorso	0:00:00,03	


[FileDati1] H:\ CARTELLE PERSONALI\Rausa Marialuisa\Articolo MIO\analisi statistiche\analisi luana\database completato maggio 2014.sav

	Fattori entro soggetticc

Misura: intensità 
tempo	Variabile dipendente	
1	i_t1	
2	i_t3	
3	i_t4	


	Fattori tra soggetti

 	N	
gruppi	1,00	11	
 	2,00	4	


	Statistiche descrittive

 	gruppi	Media	Deviazione std.	N	
Intensità	1,00	1,6100	,31925	11	
 	2,00	1,4675	,25552	4	
 	Totale	1,5720	,30174	15	
Intensità	1,00	1,4218	,21904	11	
 	2,00	1,6825	,17821	4	
 	Totale	1,4913	,23519	15	
Intensità	1,00	1,5036	,22712	11	
 	2,00	1,6350	,21424	4	
 	Totale	1,5387	,22427	15	


	Test multivariati(b)

Effetto	 	Valore	F	Ipotesi df	Gradi di libertà dell'errore	Sig.	
tempo	Traccia di Pillai	,010	,059(a)	2,000	12,000	,943	
 	Lambda di Wilks	,990	,059(a)	2,000	12,000	,943	
 	Traccia di Hotelling	,010	,059(a)	2,000	12,000	,943	
 	Radice di Roy	,010	,059(a)	2,000	12,000	,943	
tempo * gruppi	Traccia di Pillai	,244	1,938(a)	2,000	12,000	,187	
 	Lambda di Wilks	,756	1,938(a)	2,000	12,000	,187	
 	Traccia di Hotelling	,323	1,938(a)	2,000	12,000	,187	
 	Radice di Roy	,323	1,938(a)	2,000	12,000	,187	
a  Statistica esatta
b  Disegno: Intercept+gruppi 
 Disegno entro soggetti: tempo


	Test di sfericità di Mauchly(b)

Misura: intensità 
Effetto entro soggetti	W di Mauchly	Approssimazione chi-quadrato	df	Sig.	Epsilon(a)	
 	 	 	 	 	Greenhouse-Geisser	Huynh-Feldt	Limite inferiore	
tempo	,654	5,104	2	,078	,743	,881	,500	
Verifica l'ipotesi nulla per la quale la matrice di covarianza dell'errore della variabile dipendente trasformata ortonormalizzata è proporzionale a una matrice identità.
a  È possibile utilizzarlo per regolare i gradi di libertà per i test di significatività mediati. I test corretti vengono visualizzati nella tabella dei test sugli effetti entro soggetti.
b  Disegno: Intercept+gruppi 
 Disegno entro soggetti: tempo


	Test degli effetti entro soggetti

Misura: intensità 
Sorgente	 	Somma dei quadrati Tipo III	df	Media dei quadrati	F	Sig.	
tempo	Assumendo la sfericità	,006	2	,003	,056	,946	
 	Greenhouse-Geisser	,006	1,485	,004	,056	,900	
 	Huynh-Feldt	,006	1,761	,003	,056	,928	
 	Limite inferiore	,006	1,000	,006	,056	,817	
tempo * gruppi	Assumendo la sfericità	,249	2	,124	2,518	,100	
 	Greenhouse-Geisser	,249	1,485	,167	2,518	,118	
 	Huynh-Feldt	,249	1,761	,141	2,518	,108	
 	Limite inferiore	,249	1,000	,249	2,518	,137	
Errore(tempo)	Assumendo la sfericità	1,284	26	,049	 	 	
 	Greenhouse-Geisser	1,284	19,310	,066	 	 	
 	Huynh-Feldt	1,284	22,897	,056	 	 	
 	Limite inferiore	1,284	13,000	,099	 	 	


	Test dei contrasti entro soggetti

Misura: intensità 
Sorgente	tempo	Somma dei quadrati Tipo III	df	Media dei quadrati	F	Sig.	
tempo	Lineare	,005	1	,005	,084	,776	
 	Quadratico	2,75E-005	1	2,75E-005	,001	,978	
tempo * gruppi	Lineare	,110	1	,110	1,690	,216	
 	Quadratico	,139	1	,139	4,119	,063	
Errore(tempo)	Lineare	,846	13	,065	 	 	
 	Quadratico	,438	13	,034	 	 	


	Test degli effetti fra soggetti

Misura: intensità 
Variabile trasformata: Media 
Sorgente	Somma dei quadrati Tipo III	df	Media dei quadrati	F	Sig.	
Intercetta	84,940	1	84,940	952,035	,000	
gruppi	,061	1	,061	,682	,424	
Errore	1,160	13	,089	 	 	


Medie marginali attese

1. gruppi

	Stime

Misura: intensità 
gruppi	Media	Errore std.	Intervallo di confidenza 95%	
 	 	 	Limite inferiore	Limite superiore	
1,00	1,512	,052	1,399	1,624	
2,00	1,595	,086	1,409	1,781	


	Confronti a coppie

Misura: intensità 
(I) gruppi	(J) gruppi	Differenza fra medie (I-J)	Errore std.	Sig.(a)	Intervallo di confidenza per la differenza al 95%(a)	
 	 	 	 	 	Limite inferiore	Limite superiore	
1,00	2,00	-,083	,101	,424	-,301	,134	
2,00	1,00	,083	,101	,424	-,134	,301	
Basato sulle medie marginali stimate
a  Correzione per confronti multipli: Bonferroni.


	Test univariati

Misura: intensità 
 	Somma dei quadrati	df	Media dei quadrati	F	Sig.	
Contrasto	,020	1	,020	,682	,424	
Errore	,387	13	,030	 	 	
Ciascun F verifica gli effetti semplici di gruppi all'interno di ogni combinazione di livelli degli altri effetti illustrati. Questi test sono basati sui confronti a coppie indipendenti e lineari tra le medie marginali stimate.


2. tempo

	Stime

Misura: intensità 
tempo	Media	Errore std.	Intervallo di confidenza 95%	
 	 	 	Limite inferiore	Limite superiore	
1	1,539	,089	1,346	1,732	
2	1,552	,061	1,420	1,685	
3	1,569	,065	1,428	1,711	


	Confronti a coppie

Misura: intensità 
(I) tempo	(J) tempo	Differenza fra medie (I-J)	Errore std.	Sig.(a)	Intervallo di confidenza per la differenza al 95%(a)	
 	 	 	 	 	Limite inferiore	Limite superiore	
1	2	-,013	,103	1,000	-,297	,270	
 	3	-,031	,105	1,000	-,320	,259	
2	1	,013	,103	1,000	-,270	,297	
 	3	-,017	,059	1,000	-,179	,145	
3	1	,031	,105	1,000	-,259	,320	
 	2	,017	,059	1,000	-,145	,179	
Basato sulle medie marginali stimate
a  Correzione per confronti multipli: Bonferroni.


	Test multivariati

 	Valore	F	Ipotesi df	Gradi di libertà dell'errore	Sig.	
Traccia di Pillai	,010	,059(a)	2,000	12,000	,943	
Lambda di Wilks	,990	,059(a)	2,000	12,000	,943	
Traccia di Hotelling	,010	,059(a)	2,000	12,000	,943	
Radice di Roy	,010	,059(a)	2,000	12,000	,943	
Ciascun F verifica l'effetto multivariato di tempo. Questi test sono basati sui confronti a coppie indipendenti e lineari tra le medie marginali stimate.
a  Statistica esatta


3. gruppi * tempo

	Stime

Misura: intensità 
gruppi	tempo	Media	Errore std.	Intervallo di confidenza 95%	
 	 	 	 	Limite inferiore	Limite superiore	
1,00	1	1,610	,092	1,411	1,809	
 	2	1,422	,063	1,285	1,559	
 	3	1,504	,068	1,358	1,650	
2,00	1	1,468	,153	1,137	1,798	
 	2	1,683	,105	1,455	1,910	
 	3	1,635	,112	1,393	1,877	


	Confronti a coppie

Misura: intensità 
tempo	(I) gruppi	(J) gruppi	Differenza fra medie (I-J)	Errore std.	Sig.(a)	Intervallo di confidenza per la differenza al 95%(a)	
 	 	 	 	 	 	Limite inferiore	Limite superiore	
1	1,00	2,00	,143	,179	,439	-,243	,528	
 	2,00	1,00	-,143	,179	,439	-,528	,243	
2	1,00	2,00	-,261	,123	,054	-,526	,005	
 	2,00	1,00	,261	,123	,054	-,005	,526	
3	1,00	2,00	-,131	,131	,334	-,414	,151	
 	2,00	1,00	,131	,131	,334	-,151	,414	
Basato sulle medie marginali stimate
a  Correzione per confronti multipli: Bonferroni.


	Test univariati

Misura: intensità 
tempo	 	Somma dei quadrati	df	Media dei quadrati	F	Sig.	
1	Contrasto	,060	1	,060	,637	,439	
 	Errore	1,215	13	,093	 	 	
2	Contrasto	,199	1	,199	4,506	,054	
 	Errore	,575	13	,044	 	 	
3	Contrasto	,051	1	,051	1,007	,334	
 	Errore	,654	13	,050	 	 	
F verifica l'effetto di gruppi. Questo test è basato sui confronti a coppie indipendenti e lineari tra le medie marginali stimate.


4. gruppi * tempo

	Stime

Misura: intensità 
gruppi	tempo	Media	Errore std.	Intervallo di confidenza 95%	
 	 	 	 	Limite inferiore	Limite superiore	
1,00	1	1,610	,092	1,411	1,809	
 	2	1,422	,063	1,285	1,559	
 	3	1,504	,068	1,358	1,650	
2,00	1	1,468	,153	1,137	1,798	
 	2	1,683	,105	1,455	1,910	
 	3	1,635	,112	1,393	1,877	


	Confronti a coppie

Misura: intensità 
gruppi	(I) tempo	(J) tempo	Differenza fra medie (I-J)	Errore std.	Sig.(a)	Intervallo di confidenza per la differenza al 95%(a)	
 	 	 	 	 	 	Limite inferiore	Limite superiore	
1,00	1	2	,188	,107	,304	-,105	,481	
 	 	3	,106	,109	1,000	-,192	,405	
 	2	1	-,188	,107	,304	-,481	,105	
 	 	3	-,082	,061	,605	-,249	,085	
 	3	1	-,106	,109	1,000	-,405	,192	
 	 	2	,082	,061	,605	-,085	,249	
2,00	1	2	-,215	,177	,739	-,701	,271	
 	 	3	-,168	,180	1,000	-,663	,328	
 	2	1	,215	,177	,739	-,271	,701	
 	 	3	,048	,101	1,000	-,229	,324	
 	3	1	,168	,180	1,000	-,328	,663	
 	 	2	-,048	,101	1,000	-,324	,229	
Basato sulle medie marginali stimate
a  Correzione per confronti multipli: Bonferroni.


	Test multivariati

gruppi	 	Valore	F	Ipotesi df	Gradi di libertà dell'errore	Sig.	
1,00	Traccia di Pillai	,234	1,835(a)	2,000	12,000	,202	
 	Lambda di Wilks	,766	1,835(a)	2,000	12,000	,202	
 	Traccia di Hotelling	,306	1,835(a)	2,000	12,000	,202	
 	Radice di Roy	,306	1,835(a)	2,000	12,000	,202	
2,00	Traccia di Pillai	,104	,694(a)	2,000	12,000	,518	
 	Lambda di Wilks	,896	,694(a)	2,000	12,000	,518	
 	Traccia di Hotelling	,116	,694(a)	2,000	12,000	,518	
 	Radice di Roy	,116	,694(a)	2,000	12,000	,518	
Ciascun F verifica gli effetti multivariati semplici di tempo all'interno di ogni combinazione di livelli degli effetti illustrati. Questi test sono basati sui confronti a coppie indipendenti e lineari tra le medie marginali stimate.
a  Statistica esatta


Modello lineare generalizzato

	Note

Output creato	20-JUN-2014 12:25:21	
Commenti	 	
Input	Dati	H:\ CARTELLE PERSONALI\Rausa Marialuisa\Articolo MIO\analisi statistiche\analisi luana\database completato maggio 2014.sav	
 	File di dati attivo	FileDati1	
 	Filtro	<nessuno>	
 	Peso	<nessuno>	
 	Distingui	<nessuno>	
 	N. di righe nel file dati di lavoro	31	
Gestione valori mancanti	Definizione di valore mancante	I valori mancanti definiti dall'utente vengono considerati mancanti.	
 	Casi utilizzati	Le statistiche sono basate su tutti i casi con dati validi di tutte le variabili del modello.	
Sintassi	GLM d_T1 d_T3 d_T4 BY gruppi
  /WSFACTOR=tempo 3 Polynomial
  /MEASURE=durata
  /METHOD=SSTYPE(3)
  /EMMEANS=TABLES(gruppi) COMPARE ADJ(BONFERRONI)
  /EMMEANS=TABLES(tempo) COMPARE ADJ(BONFERRONI)
  /EMMEANS=TABLES(gruppi*tempo) COMPARE(gruppi) ADJ(BONFERRONI)
 /EMMEANS=TABLES(gruppi*tempo) COMPARE(tempo) ADJ(BONFERRONI) /PRINT=DESCRIPTIVE
  /CRITERIA=ALPHA(.05)
  /WSDESIGN=tempo
  /DESIGN=gruppi.
	
Risorse	Tempo trascorso	0:00:00,03	


[FileDati1] H:\ CARTELLE PERSONALI\Rausa Marialuisa\Articolo MIO\analisi statistiche\analisi luana\database completato maggio 2014.sav

	Fattori entro soggetticc

Misura: durata 
tempo	Variabile dipendente	
1	d_t1	
2	d_t3	
3	d_t4	


	Fattori tra soggetti

 	N	
gruppi	1,00	10	
 	2,00	4	


	Statistiche descrittive

 	gruppi	Media	Deviazione std.	N	
Durata	1,00	6,1460	3,15636	10	
 	2,00	5,1475	4,34765	4	
 	Totale	5,8607	3,38797	14	
Durata	1,00	6,6390	3,36482	10	
 	2,00	8,7725	6,28549	4	
 	Totale	7,2486	4,23743	14	
Durata	1,00	6,2520	3,45998	10	
 	2,00	8,7975	5,92206	4	
 	Totale	6,9793	4,21963	14	


	Test multivariati(b)

Effetto	 	Valore	F	Ipotesi df	Gradi di libertà dell'errore	Sig.	
tempo	Traccia di Pillai	,481	5,103(a)	2,000	11,000	,027	
 	Lambda di Wilks	,519	5,103(a)	2,000	11,000	,027	
 	Traccia di Hotelling	,928	5,103(a)	2,000	11,000	,027	
 	Radice di Roy	,928	5,103(a)	2,000	11,000	,027	
tempo * gruppi	Traccia di Pillai	,382	3,405(a)	2,000	11,000	,071	
 	Lambda di Wilks	,618	3,405(a)	2,000	11,000	,071	
 	Traccia di Hotelling	,619	3,405(a)	2,000	11,000	,071	
 	Radice di Roy	,619	3,405(a)	2,000	11,000	,071	
a  Statistica esatta
b  Disegno: Intercept+gruppi 
 Disegno entro soggetti: tempo


	Test di sfericità di Mauchly(b)

Misura: durata 
Effetto entro soggetti	W di Mauchly	Approssimazione chi-quadrato	df	Sig.	Epsilon(a)	
 	 	 	 	 	Greenhouse-Geisser	Huynh-Feldt	Limite inferiore	
tempo	,921	,902	2	,637	,927	1,000	,500	
Verifica l'ipotesi nulla per la quale la matrice di covarianza dell'errore della variabile dipendente trasformata ortonormalizzata è proporzionale a una matrice identità.
a  È possibile utilizzarlo per regolare i gradi di libertà per i test di significatività mediati. I test corretti vengono visualizzati nella tabella dei test sugli effetti entro soggetti.
b  Disegno: Intercept+gruppi 
 Disegno entro soggetti: tempo


	Test degli effetti entro soggetti

Misura: durata 
Sorgente	 	Somma dei quadrati Tipo III	df	Media dei quadrati	F	Sig.	
tempo	Assumendo la sfericità	29,711	2	14,855	6,331	,006	
 	Greenhouse-Geisser	29,711	1,854	16,024	6,331	,008	
 	Huynh-Feldt	29,711	2,000	14,855	6,331	,006	
 	Limite inferiore	29,711	1,000	29,711	6,331	,027	
tempo * gruppi	Assumendo la sfericità	21,466	2	10,733	4,574	,021	
 	Greenhouse-Geisser	21,466	1,854	11,577	4,574	,024	
 	Huynh-Feldt	21,466	2,000	10,733	4,574	,021	
 	Limite inferiore	21,466	1,000	21,466	4,574	,054	
Errore(tempo)	Assumendo la sfericità	56,317	24	2,347	 	 	
 	Greenhouse-Geisser	56,317	22,249	2,531	 	 	
 	Huynh-Feldt	56,317	24,000	2,347	 	 	
 	Limite inferiore	56,317	12,000	4,693	 	 	


	Test dei contrasti entro soggetti

Misura: durata 
Sorgente	tempo	Somma dei quadrati Tipo III	df	Media dei quadrati	F	Sig.	
tempo	Lineare	20,154	1	20,154	6,813	,023	
 	Quadratico	9,557	1	9,557	5,509	,037	
tempo * gruppi	Lineare	17,943	1	17,943	6,065	,030	
 	Quadratico	3,523	1	3,523	2,031	,180	
Errore(tempo)	Lineare	35,499	12	2,958	 	 	
 	Quadratico	20,818	12	1,735	 	 	


	Test degli effetti fra soggetti

Misura: durata 
Variabile trasformata: Media 
Sorgente	Somma dei quadrati Tipo III	df	Media dei quadrati	F	Sig.	
Intercetta	1660,417	1	1660,417	38,066	,000	
gruppi	12,901	1	12,901	,296	,597	
Errore	523,428	12	43,619	 	 	


Medie marginali attese

1. gruppi

	Stime

Misura: durata 
gruppi	Media	Errore std.	Intervallo di confidenza 95%	
 	 	 	Limite inferiore	Limite superiore	
1,00	6,346	1,206	3,718	8,973	
2,00	7,573	1,907	3,418	11,727	


	Confronti a coppie

Misura: durata 
(I) gruppi	(J) gruppi	Differenza fra medie (I-J)	Errore std.	Sig.(a)	Intervallo di confidenza per la differenza al 95%(a)	
 	 	 	 	 	Limite inferiore	Limite superiore	
1,00	2,00	-1,227	2,256	,597	-6,142	3,688	
2,00	1,00	1,227	2,256	,597	-3,688	6,142	
Basato sulle medie marginali stimate
a  Correzione per confronti multipli: Bonferroni.


	Test univariati

Misura: durata 
 	Somma dei quadrati	df	Media dei quadrati	F	Sig.	
Contrasto	4,300	1	4,300	,296	,597	
Errore	174,476	12	14,540	 	 	
Ciascun F verifica gli effetti semplici di gruppi all'interno di ogni combinazione di livelli degli altri effetti illustrati. Questi test sono basati sui confronti a coppie indipendenti e lineari tra le medie marginali stimate.


2. tempo

	Stime

Misura: durata 
tempo	Media	Errore std.	Intervallo di confidenza 95%	
 	 	 	Limite inferiore	Limite superiore	
1	5,647	1,033	3,396	7,898	
2	7,706	1,268	4,944	10,468	
3	7,525	1,246	4,810	10,240	


	Confronti a coppie

Misura: durata 
(I) tempo	(J) tempo	Differenza fra medie (I-J)	Errore std.	Sig.(a)	Intervallo di confidenza per la differenza al 95%(a)	
 	 	 	 	 	Limite inferiore	Limite superiore	
1	2	-2,059(*)	,628	,020	-3,804	-,314	
 	3	-1,878	,720	,068	-3,878	,122	
2	1	2,059(*)	,628	,020	,314	3,804	
 	3	,181	,566	1,000	-1,392	1,754	
3	1	1,878	,720	,068	-,122	3,878	
 	2	-,181	,566	1,000	-1,754	1,392	
Basato sulle medie marginali stimate
*  La differenza fra medie è significativa al livello ,05
a  Correzione per confronti multipli: Bonferroni.


	Test multivariati

 	Valore	F	Ipotesi df	Gradi di libertà dell'errore	Sig.	
Traccia di Pillai	,481	5,103(a)	2,000	11,000	,027	
Lambda di Wilks	,519	5,103(a)	2,000	11,000	,027	
Traccia di Hotelling	,928	5,103(a)	2,000	11,000	,027	
Radice di Roy	,928	5,103(a)	2,000	11,000	,027	
Ciascun F verifica l'effetto multivariato di tempo. Questi test sono basati sui confronti a coppie indipendenti e lineari tra le medie marginali stimate.
a  Statistica esatta


3. gruppi * tempo

	Stime

Misura: durata 
gruppi	tempo	Media	Errore std.	Intervallo di confidenza 95%	
 	 	 	 	Limite inferiore	Limite superiore	
1,00	1	6,146	1,104	3,740	8,552	
 	2	6,639	1,355	3,686	9,592	
 	3	6,252	1,332	3,349	9,155	
2,00	1	5,148	1,746	1,343	8,952	
 	2	8,773	2,143	4,103	13,442	
 	3	8,798	2,106	4,208	13,387	


	Confronti a coppie

Misura: durata 
tempo	(I) gruppi	(J) gruppi	Differenza fra medie (I-J)	Errore std.	Sig.(a)	Intervallo di confidenza per la differenza al 95%(a)	
 	 	 	 	 	 	Limite inferiore	Limite superiore	
1	1,00	2,00	,999	2,066	,638	-3,503	5,500	
 	2,00	1,00	-,999	2,066	,638	-5,500	3,503	
2	1,00	2,00	-2,134	2,536	,417	-7,658	3,391	
 	2,00	1,00	2,134	2,536	,417	-3,391	7,658	
3	1,00	2,00	-2,546	2,492	,327	-7,976	2,885	
 	2,00	1,00	2,546	2,492	,327	-2,885	7,976	
Basato sulle medie marginali stimate
a  Correzione per confronti multipli: Bonferroni.


	Test univariati

Misura: durata 
tempo	 	Somma dei quadrati	df	Media dei quadrati	F	Sig.	
1	Contrasto	2,849	1	2,849	,234	,638	
 	Errore	146,370	12	12,197	 	 	
2	Contrasto	13,005	1	13,005	,708	,417	
 	Errore	220,420	12	18,368	 	 	
3	Contrasto	18,513	1	18,513	1,043	,327	
 	Errore	212,955	12	17,746	 	 	
F verifica l'effetto di gruppi. Questo test è basato sui confronti a coppie indipendenti e lineari tra le medie marginali stimate.


4. gruppi * tempo

	Stime

Misura: durata 
gruppi	tempo	Media	Errore std.	Intervallo di confidenza 95%	
 	 	 	 	Limite inferiore	Limite superiore	
1,00	1	6,146	1,104	3,740	8,552	
 	2	6,639	1,355	3,686	9,592	
 	3	6,252	1,332	3,349	9,155	
2,00	1	5,148	1,746	1,343	8,952	
 	2	8,773	2,143	4,103	13,442	
 	3	8,798	2,106	4,208	13,387	


	Confronti a coppie

Misura: durata 
gruppi	(I) tempo	(J) tempo	Differenza fra medie (I-J)	Errore std.	Sig.(a)	Intervallo di confidenza per la differenza al 95%(a)	
 	 	 	 	 	 	Limite inferiore	Limite superiore	
1,00	1	2	-,493	,671	1,000	-2,358	1,372	
 	 	3	-,106	,769	1,000	-2,244	2,032	
 	2	1	,493	,671	1,000	-1,372	2,358	
 	 	3	,387	,605	1,000	-1,295	2,069	
 	3	1	,106	,769	1,000	-2,032	2,244	
 	 	2	-,387	,605	1,000	-2,069	1,295	
2,00	1	2	-3,625(*)	1,061	,015	-6,574	-,676	
 	 	3	-3,650(*)	1,216	,033	-7,030	-,270	
 	2	1	3,625(*)	1,061	,015	,676	6,574	
 	 	3	-,025	,957	1,000	-2,684	2,634	
 	3	1	3,650(*)	1,216	,033	,270	7,030	
 	 	2	,025	,957	1,000	-2,634	2,684	
Basato sulle medie marginali stimate
*  La differenza fra medie è significativa al livello ,05
a  Correzione per confronti multipli: Bonferroni.


	Test multivariati

gruppi	 	Valore	F	Ipotesi df	Gradi di libertà dell'errore	Sig.	
1,00	Traccia di Pillai	,059	,342(a)	2,000	11,000	,718	
 	Lambda di Wilks	,941	,342(a)	2,000	11,000	,718	
 	Traccia di Hotelling	,062	,342(a)	2,000	11,000	,718	
 	Radice di Roy	,062	,342(a)	2,000	11,000	,718	
2,00	Traccia di Pillai	,514	5,819(a)	2,000	11,000	,019	
 	Lambda di Wilks	,486	5,819(a)	2,000	11,000	,019	
 	Traccia di Hotelling	1,058	5,819(a)	2,000	11,000	,019	
 	Radice di Roy	1,058	5,819(a)	2,000	11,000	,019	
Ciascun F verifica gli effetti multivariati semplici di tempo all'interno di ogni combinazione di livelli degli effetti illustrati. Questi test sono basati sui confronti a coppie indipendenti e lineari tra le medie marginali stimate.
a  Statistica esatta


Modello lineare generalizzato

	Note

Output creato	20-JUN-2014 12:25:57	
Commenti	 	
Input	Dati	H:\ CARTELLE PERSONALI\Rausa Marialuisa\Articolo MIO\analisi statistiche\analisi luana\database completato maggio 2014.sav	
 	File di dati attivo	FileDati1	
 	Filtro	<nessuno>	
 	Peso	<nessuno>	
 	Distingui	<nessuno>	
 	N. di righe nel file dati di lavoro	31	
Gestione valori mancanti	Definizione di valore mancante	I valori mancanti definiti dall'utente vengono considerati mancanti.	
 	Casi utilizzati	Le statistiche sono basate su tutti i casi con dati validi di tutte le variabili del modello.	
Sintassi	GLM n_anal_T1 n_anal_T3 n_anal_T4 BY gruppi
  /WSFACTOR=tempo 3 Polynomial
  /MEASURE=analgesici
  /METHOD=SSTYPE(3)
  /EMMEANS=TABLES(gruppi) COMPARE ADJ(BONFERRONI)
  /EMMEANS=TABLES(tempo) COMPARE ADJ(BONFERRONI)
  /EMMEANS=TABLES(gruppi*tempo) COMPARE(gruppi) ADJ(BONFERRONI)
 /EMMEANS=TABLES(gruppi*tempo) COMPARE(tempo) ADJ(BONFERRONI) /PRINT=DESCRIPTIVE
  /CRITERIA=ALPHA(.05)
  /WSDESIGN=tempo
  /DESIGN=gruppi.
	
Risorse	Tempo trascorso	0:00:00,03	


[FileDati1] H:\ CARTELLE PERSONALI\Rausa Marialuisa\Articolo MIO\analisi statistiche\analisi luana\database completato maggio 2014.sav

	Fattori entro soggetticc

Misura: analgesici 
tempo	Variabile dipendente	
1	n_anal_t1	
2	n_anal_t3	
3	n_anal_t4	


	Fattori tra soggetti

 	N	
gruppi	1,00	15	
 	2,00	12	


	Statistiche descrittive

 	gruppi	Media	Deviazione std.	N	
analgesici	1,00	21,2667	9,40719	15	
 	2,00	26,6250	18,70479	12	
 	Totale	23,6481	14,24903	27	
analgesici	1,00	11,9333	4,86190	15	
 	2,00	27,0417	20,84407	12	
 	Totale	18,6481	15,97102	27	
analgesici	1,00	12,4000	5,50065	15	
 	2,00	27,1667	26,40191	12	
 	Totale	18,9630	19,16022	27	


	Test multivariati(b)

Effetto	 	Valore	F	Ipotesi df	Gradi di libertà dell'errore	Sig.	
tempo	Traccia di Pillai	,223	3,453(a)	2,000	24,000	,048	
 	Lambda di Wilks	,777	3,453(a)	2,000	24,000	,048	
 	Traccia di Hotelling	,288	3,453(a)	2,000	24,000	,048	
 	Radice di Roy	,288	3,453(a)	2,000	24,000	,048	
tempo * gruppi	Traccia di Pillai	,256	4,119(a)	2,000	24,000	,029	
 	Lambda di Wilks	,744	4,119(a)	2,000	24,000	,029	
 	Traccia di Hotelling	,343	4,119(a)	2,000	24,000	,029	
 	Radice di Roy	,343	4,119(a)	2,000	24,000	,029	
a  Statistica esatta
b  Disegno: Intercept+gruppi 
 Disegno entro soggetti: tempo


	Test di sfericità di Mauchly(b)

Misura: analgesici 
Effetto entro soggetti	W di Mauchly	Approssimazione chi-quadrato	df	Sig.	Epsilon(a)	
 	 	 	 	 	Greenhouse-Geisser	Huynh-Feldt	Limite inferiore	
tempo	,703	8,452	2	,015	,771	,845	,500	
Verifica l'ipotesi nulla per la quale la matrice di covarianza dell'errore della variabile dipendente trasformata ortonormalizzata è proporzionale a una matrice identità.
a  È possibile utilizzarlo per regolare i gradi di libertà per i test di significatività mediati. I test corretti vengono visualizzati nella tabella dei test sugli effetti entro soggetti.
b  Disegno: Intercept+gruppi 
 Disegno entro soggetti: tempo


	Test degli effetti entro soggetti

Misura: analgesici 
Sorgente	 	Somma dei quadrati Tipo III	df	Media dei quadrati	F	Sig.	
tempo	Assumendo la sfericità	331,473	2	165,736	4,516	,016	
 	Greenhouse-Geisser	331,473	1,542	214,932	4,516	,025	
 	Huynh-Feldt	331,473	1,690	196,156	4,516	,022	
 	Limite inferiore	331,473	1,000	331,473	4,516	,044	
tempo * gruppi	Assumendo la sfericità	408,213	2	204,107	5,561	,007	
 	Greenhouse-Geisser	408,213	1,542	264,692	5,561	,012	
 	Huynh-Feldt	408,213	1,690	241,569	5,561	,010	
 	Limite inferiore	408,213	1,000	408,213	5,561	,026	
Errore(tempo)	Assumendo la sfericità	1835,169	50	36,703	 	 	
 	Greenhouse-Geisser	1835,169	38,556	47,598	 	 	
 	Huynh-Feldt	1835,169	42,246	43,440	 	 	
 	Limite inferiore	1835,169	25,000	73,407	 	 	


	Test dei contrasti entro soggetti

Misura: analgesici 
Sorgente	tempo	Somma dei quadrati Tipo III	df	Media dei quadrati	F	Sig.	
tempo	Lineare	231,019	1	231,019	4,285	,049	
 	Quadratico	100,454	1	100,454	5,152	,032	
tempo * gruppi	Lineare	295,056	1	295,056	5,473	,028	
 	Quadratico	113,157	1	113,157	5,804	,024	
Errore(tempo)	Lineare	1347,731	25	53,909	 	 	
 	Quadratico	487,438	25	19,498	 	 	


	Test degli effetti fra soggetti

Misura: analgesici 
Variabile trasformata: Media 
Sorgente	Somma dei quadrati Tipo III	df	Media dei quadrati	F	Sig.	
Intercetta	35523,084	1	35523,084	53,974	,000	
gruppi	2758,640	1	2758,640	4,192	,051	
Errore	16453,756	25	658,150	 	 	


Medie marginali attese

1. gruppi

	Stime

Misura: analgesici 
gruppi	Media	Errore std.	Intervallo di confidenza 95%	
 	 	 	Limite inferiore	Limite superiore	
1,00	15,200	3,824	7,324	23,076	
2,00	26,944	4,276	18,138	35,750	


	Confronti a coppie

Misura: analgesici 
(I) gruppi	(J) gruppi	Differenza fra medie (I-J)	Errore std.	Sig.(a)	Intervallo di confidenza per la differenza al 95%(a)	
 	 	 	 	 	Limite inferiore	Limite superiore	
1,00	2,00	-11,744	5,737	,051	-23,559	,070	
2,00	1,00	11,744	5,737	,051	-,070	23,559	
Basato sulle medie marginali stimate
a  Correzione per confronti multipli: Bonferroni.


	Test univariati

Misura: analgesici 
 	Somma dei quadrati	df	Media dei quadrati	F	Sig.	
Contrasto	919,547	1	919,547	4,192	,051	
Errore	5484,585	25	219,383	 	 	
Ciascun F verifica gli effetti semplici di gruppi all'interno di ogni combinazione di livelli degli altri effetti illustrati. Questi test sono basati sui confronti a coppie indipendenti e lineari tra le medie marginali stimate.


2. tempo

	Stime

Misura: analgesici 
tempo	Media	Errore std.	Intervallo di confidenza 95%	
 	 	 	Limite inferiore	Limite superiore	
1	23,946	2,762	18,256	29,635	
2	19,488	2,769	13,785	25,190	
3	19,783	3,484	12,608	26,958	


	Confronti a coppie

Misura: analgesici 
(I) tempo	(J) tempo	Differenza fra medie (I-J)	Errore std.	Sig.(a)	Intervallo di confidenza per la differenza al 95%(a)	
 	 	 	 	 	Limite inferiore	Limite superiore	
1	2	4,458(*)	1,664	,039	,188	8,728	
 	3	4,163	2,011	,147	-,997	9,322	
2	1	-4,458(*)	1,664	,039	-8,728	-,188	
 	3	-,296	1,202	1,000	-3,381	2,789	
3	1	-4,163	2,011	,147	-9,322	,997	
 	2	,296	1,202	1,000	-2,789	3,381	
Basato sulle medie marginali stimate
*  La differenza fra medie è significativa al livello ,05
a  Correzione per confronti multipli: Bonferroni.


	Test multivariati

 	Valore	F	Ipotesi df	Gradi di libertà dell'errore	Sig.	
Traccia di Pillai	,223	3,453(a)	2,000	24,000	,048	
Lambda di Wilks	,777	3,453(a)	2,000	24,000	,048	
Traccia di Hotelling	,288	3,453(a)	2,000	24,000	,048	
Radice di Roy	,288	3,453(a)	2,000	24,000	,048	
Ciascun F verifica l'effetto multivariato di tempo. Questi test sono basati sui confronti a coppie indipendenti e lineari tra le medie marginali stimate.
a  Statistica esatta


3. gruppi * tempo

	Stime

Misura: analgesici 
gruppi	tempo	Media	Errore std.	Intervallo di confidenza 95%	
 	 	 	 	Limite inferiore	Limite superiore	
1,00	1	21,267	3,683	13,681	28,853	
 	2	11,933	3,691	4,331	19,536	
 	3	12,400	4,645	2,833	21,967	
2,00	1	26,625	4,118	18,144	35,106	
 	2	27,042	4,127	18,542	35,542	
 	3	27,167	5,193	16,471	37,863	


	Confronti a coppie

Misura: analgesici 
tempo	(I) gruppi	(J) gruppi	Differenza fra medie (I-J)	Errore std.	Sig.(a)	Intervallo di confidenza per la differenza al 95%(a)	
 	 	 	 	 	 	Limite inferiore	Limite superiore	
1	1,00	2,00	-5,358	5,525	,341	-16,737	6,020	
 	2,00	1,00	5,358	5,525	,341	-6,020	16,737	
2	1,00	2,00	-15,108(*)	5,537	,011	-26,512	-3,704	
 	2,00	1,00	15,108(*)	5,537	,011	3,704	26,512	
3	1,00	2,00	-14,767(*)	6,968	,044	-29,117	-,417	
 	2,00	1,00	14,767(*)	6,968	,044	,417	29,117	
Basato sulle medie marginali stimate
*  La differenza fra medie è significativa al livello ,05
a  Correzione per confronti multipli: Bonferroni.


	Test univariati

Misura: analgesici 
tempo	 	Somma dei quadrati	df	Media dei quadrati	F	Sig.	
1	Contrasto	191,412	1	191,412	,941	,341	
 	Errore	5087,496	25	203,500	 	 	
2	Contrasto	1521,745	1	1521,745	7,445	,011	
 	Errore	5110,163	25	204,407	 	 	
3	Contrasto	1453,696	1	1453,696	4,492	,044	
 	Errore	8091,267	25	323,651	 	 	
F verifica l'effetto di gruppi. Questo test è basato sui confronti a coppie indipendenti e lineari tra le medie marginali stimate.


4. gruppi * tempo

	Stime

Misura: analgesici 
gruppi	tempo	Media	Errore std.	Intervallo di confidenza 95%	
 	 	 	 	Limite inferiore	Limite superiore	
1,00	1	21,267	3,683	13,681	28,853	
 	2	11,933	3,691	4,331	19,536	
 	3	12,400	4,645	2,833	21,967	
2,00	1	26,625	4,118	18,144	35,106	
 	2	27,042	4,127	18,542	35,542	
 	3	27,167	5,193	16,471	37,863	


	Confronti a coppie

Misura: analgesici 
gruppi	(I) tempo	(J) tempo	Differenza fra medie (I-J)	Errore std.	Sig.(a)	Intervallo di confidenza per la differenza al 95%(a)	
 	 	 	 	 	 	Limite inferiore	Limite superiore	
1,00	1	2	9,333(*)	2,219	,001	3,640	15,027	
 	 	3	8,867(*)	2,681	,009	1,987	15,746	
 	2	1	-9,333(*)	2,219	,001	-15,027	-3,640	
 	 	3	-,467	1,603	1,000	-4,580	3,647	
 	3	1	-8,867(*)	2,681	,009	-15,746	-1,987	
 	 	2	,467	1,603	1,000	-3,647	4,580	
2,00	1	2	-,417	2,481	1,000	-6,782	5,949	
 	 	3	-,542	2,997	1,000	-8,233	7,150	
 	2	1	,417	2,481	1,000	-5,949	6,782	
 	 	3	-,125	1,792	1,000	-4,724	4,474	
 	3	1	,542	2,997	1,000	-7,150	8,233	
 	 	2	,125	1,792	1,000	-4,474	4,724	
Basato sulle medie marginali stimate
*  La differenza fra medie è significativa al livello ,05
a  Correzione per confronti multipli: Bonferroni.


	Test multivariati

gruppi	 	Valore	F	Ipotesi df	Gradi di libertà dell'errore	Sig.	
1,00	Traccia di Pillai	,415	8,499(a)	2,000	24,000	,002	
 	Lambda di Wilks	,585	8,499(a)	2,000	24,000	,002	
 	Traccia di Hotelling	,708	8,499(a)	2,000	24,000	,002	
 	Radice di Roy	,708	8,499(a)	2,000	24,000	,002	
2,00	Traccia di Pillai	,001	,016(a)	2,000	24,000	,984	
 	Lambda di Wilks	,999	,016(a)	2,000	24,000	,984	
 	Traccia di Hotelling	,001	,016(a)	2,000	24,000	,984	
 	Radice di Roy	,001	,016(a)	2,000	24,000	,984	
Ciascun F verifica gli effetti multivariati semplici di tempo all'interno di ogni combinazione di livelli degli effetti illustrati. Questi test sono basati sui confronti a coppie indipendenti e lineari tra le medie marginali stimate.
a  Statistica esatta
