# Supplementary material for: Biofeedback in the prophylactic treatment of medication overuse headache: a pilot randomized controlled trial
Source: J Headache Pain. 2016 Sep 22;17(1):87. doi: 10.1186/s10194-016-0679-9 (PMC5031562; doi:10.1186/s10194-016-0679-9)
Supplement: Additional file 3: — Statistical analysis on questionnaires variables. (PDF 281 kb) [file 10194_2016_679_MOESM3_ESM.pdf]

odello lineare generalizzato

Note

ıtput creato		19-JUN-2014 11:25:21
ɔmmenti		
ɔut	Dati	H:\• CARTELLE PERSONALI\Rausa Marialuisa\Articolo MIO\analisi statistiche\analisi luana\database completato maggio 2014.sav
	File di dati attivo	FileDati1
	Filtro	<nessuno>
	Peso	<nessuno>
	Distingui	<nessuno>
	N. di righe nel file dati di lavoro	31
əstione valori ancanti	Definizione di valore mancante	I valori mancanti definiti dall'utente vengono considerati mancanti.
	Casi utilizzati	Le statistiche sono basate su tutti i casi con dati validi di tutte le variabili del modello.
ntassi		GLM FSkpr_T1 FSkpr_T3 FSkpr_T4 BY gruppi<br>/WSFACTOR=tempo 3 Polynomial<br>/MEASURE=pr<br>/METHOD=SSTYPE(3)<br>/EMMEANS=TABLES(gruppi) COMPARE ADJ(BONFERRONI)<br>/EMMEANS=TABLES(tempo) COMPARE ADJ(BONFERRONI)<br>/EMMEANS=TABLES(gruppi*tempo) COMPARE(gruppi) ADJ(BONFERRONI)<br>/EMMEANS=TABLES(gruppi*tempo) COMPARE(tempo) ADJ(BONFERRONI)<br>/PRINT=DESCRIPTIVE<br>/CRITERIA=ALPHA(.05)<br>/WSDESIGN=tempo<br>/DESIGN=gruppi.
sorse	Tempo trascorso	0:00:00,03

```
ileDati1] H:\• CARTELLE PERSONALI\Rausa Marialuisa\Articolo MIO\analisi
atistiche\analisi luana\database completato maggio 2014.sav
```

Fattori entro soggetticc

ura: pr

npo	Variabile dipendente
	FSkpr_t1
	FSkpr_t3
	FSkpr_t4

Fattori tra soggetti

		N
uppi	1,00	14
	2,00	11

Statistiche descrittive

	gruppi	Media	Deviazione std.	N
;kpr_t1	1,00	2,5207	,61216	14
	2,00	2,5818	,94358	11
	Totale	2,5476	,75823	25
;kpr_t3	1,00	2,6929	,65172	14
	2,00	2,8655	,86738	11
	Totale	2,7688	,74242	25
;kpr_t4	1,00	2,7171	,73545	14
	2,00	2,7291	,73180	11
	Totale	2,7224	,71844	25

Test multivariati(b)

fetto		Valore	F	Ipotesi df	Gradi di libertà dell'errore	Sig.
npo	Traccia di Pillai	,095	1,161(a)	2,000	22,000	,332
	Lambda di Wilks	,905	1,161(a)	2,000	22,000	,332
	Traccia di Hotelling	,106	1,161(a)	2,000	22,000	,332
	Radice di Roy	,106	1,161(a)	2,000	22,000	,332
npo * gruppi	Traccia di Pillai	,023	,264(a)	2,000	22,000	,770
	Lambda di Wilks	,977	,264(a)	2,000	22,000	,770
	Traccia di Hotelling	,024	,264(a)	2,000	22,000	,770
	Radice di Roy	,024	,264(a)	2,000	22,000	,770

;tatistica esatta
)isegno: Intercept+gruppi
;egno entro soggetti: tempo

Test di sfericità di Mauchly(b)

ura: pr

fetto entro soggetti	W di Mauchly	Approssimazione chi-quadrato	df	Sig.	Epsilon(a)		
					Greenhouse-Geisser	Huynh-Feldt	Limite inferiore
npo	,877	2,890	2	,236	,890	1,000	,500

ifica l'ipotesi nulla per la quale la matrice di covarianza dell'errore della variabile dipendente trasformata ortonormalizzata è

porzionale a una matrice identità.
: possibile utilizzarlo per regolare i gradi di libertà per i test di significatività mediati. I test corretti vengono visualizzati nella tabella dei
: sugli effetti entro soggetti.
)isegno: Intercept+gruppi
segno entro soggetti: tempo

Test degli effetti entro soggetti

ura: pr

rgente		Somma dei quadrati Tipo III	df	Media dei quadrati	F	Sig.
npo	Assumendo la sfericità	,695	2	,347	1,519	,230
	Greenhouse-Geisser	,695	1,781	,390	1,519	,232
	Huynh-Feldt	,695	2,000	,347	1,519	,230
	Limite inferiore	,695	1,000	,695	1,519	,230
npo * gruppi	Assumendo la sfericità	,083	2	,042	,182	,834
	Greenhouse-Geisser	,083	1,781	,047	,182	,809
	Huynh-Feldt	,083	2,000	,042	,182	,834
	Limite inferiore	,083	1,000	,083	,182	,673
rore(tempo)	Assumendo la sfericità	10,521	46	,229		
	Greenhouse-Geisser	10,521	40,959	,257		
	Huynh-Feldt	10,521	46,000	,229		
	Limite inferiore	10,521	23,000	,457		

Test dei contrasti entro soggetti

ura: pr

rgente	tempo	Somma dei quadrati Tipo III	df	Media dei quadrati	F	Sig.
npo	Lineare	,364	1	,364	1,329	,261
	Quadratico	,331	1	,331	1,803	,192
npo * gruppi	Lineare	,007	1	,007	,027	,870
	Quadratico	,076	1	,076	,414	,526
rore(tempo)	Lineare	6,298	23	,274		
	Quadratico	4,223	23	,184		

Test degli effetti fra soggetti

ura: pr
iabile trasformata: Media

rgente	Somma dei quadrati Tipo III	df	Media dei quadrati	F	Sig.
tercetta	532,713	1	532,713	427,125	,000
uppi	,124	1	,124	,099	,755
rore	28,686	23	1,247		

edie marginali attese

gruppi

Stime

ura: pr

uppi	Media	Errore std.	Intervallo di confidenza 95%	
			Limite inferiore	Limite superiore
)0	2,644	,172	2,287	3,000
)0	2,725	,194	2,323	3,128

Confronti a coppie

ura: pr

gruppi	(J) gruppi	Differenza fra medie (I-J)	Errore std.	Sig.(a)	Intervallo di confidenza per la differenza al 95%(a)	
					Limite inferiore	Limite superiore
)0	2,00	-,082	,260	,755	-,619	,456
)0	1,00	,082	,260	,755	-,456	,619

;ato sulle medie marginali stimate
:orrezione per confronti multipli: Bonferroni.

Test univariati

ura: pr

	Somma dei quadrati	df	Media dei quadrati	F	Sig.
ɔntrasto	,041	1	,041	,099	,755
rore	9,562	23	,416		

scun F verifica gli effetti semplici di gruppi all'interno di ogni combinazione di livelli degli altri effetti illustrati. Questi test sono basati confronti a coppie indipendenti e lineari tra le medie marginali stimate.

tempo

Stime

ura: pr

mpo	Media	Errore std.	Intervallo di confidenza 95%	
			Limite inferiore	Limite superiore
	2,551	,156	2,229	2,874
	2,779	,152	2,465	3,093
	2,723	,148	2,417	3,029

Confronti a coppie

ura: pr

tempo	(J) tempo	Differenza fra medie (I-J)	Errore std.	Sig.(a)	Intervallo di confidenza per la differenza al 95%(a) Limite inferiore	Limite superiore
	2	-,228	,146	,399	-,606	,150
	3	-,172	,149	,783	-,557	,213
	1	,228	,146	,399	-,150	,606
	3	,056	,110	1,000	-,228	,340
	1	,172	,149	,783	-,213	,557
	2	-,056	,110	1,000	-,340	,228

ɜato sulle medie marginali stimate
Ↄorrezione per confronti multipli: Bonferroni.

Test multivariati

	Valore	F	Ipotesi df	Gradi di libertà dell'errore	Sig.
accia di Pillai	,095	1,161(a)	2,000	22,000	,332
ımbda di Wilks	,905	1,161(a)	2,000	22,000	,332
accia di Hotelling	,106	1,161(a)	2,000	22,000	,332
ɜdice di Roy	,106	1,161(a)	2,000	22,000	,332

scun F verifica l'effetto multivariato di tempo. Questi test sono basati sui confronti a coppie indipendenti e lineari tra le medie rginali stimate.
ꜱtatistica esatta

gruppi * tempo

Stime

ura: pr

uppi	tempo	Media	Errore std.	Intervallo di confidenza 95% Limite inferiore	Limite superiore
00	1	2,521	,207	2,093	2,949
	2	2,693	,201	2,276	3,109
	3	2,717	,196	2,311	3,123
00	1	2,582	,233	2,099	3,065
	2	2,865	,227	2,396	3,335
	3	2,729	,221	2,271	3,187

Confronti a coppie

ura: pr

npo	(I) gruppi	(J) gruppi	Differenza fra medie (I-J)	Errore std.	Sig.(a)	Intervallo di confidenza per la differenza al 95%(a)

					Limite inferiore	Limite superiore
1,00	2,00	-,061	,312	,846	-,706	,584
2,00	1,00	,061	,312	,846	-,584	,706
1,00	2,00	-,173	,303	,575	-,800	,455
2,00	1,00	,173	,303	,575	-,455	,800
1,00	2,00	-,012	,296	,968	-,624	,600
2,00	1,00	,012	,296	,968	-,600	,624

sato sulle medie marginali stimate
Correzione per confronti multipli: Bonferroni.

Test univariati

ura: pr

mpo		Somma dei quadrati	df	Media dei quadrati	F	Sig.
	Contrasto	,023	1	,023	,038	,846
	Errore	13,775	23	,599		
	Contrasto	,184	1	,184	,324	,575
	Errore	13,045	23	,567		
	Contrasto	,001	1	,001	,002	,968
	Errore	12,387	23	,539		

erifica l'effetto di gruppi. Questo test è basato sui confronti a coppie indipendenti e lineari tra le medie marginali stimate.

gruppi * tempo

Stime

ura: pr

				Intervallo di confidenza 95%	
uppi	tempo	Media	Errore std.	Limite inferiore	Limite superiore
00	1	2,521	,207	2,093	2,949
	2	2,693	,201	2,276	3,109
	3	2,717	,196	2,311	3,123
00	1	2,582	,233	2,099	3,065
	2	2,865	,227	2,396	3,335
	3	2,729	,221	2,271	3,187

Confronti a coppie

ura: pr

						Intervallo di confidenza per la differenza al 95%(a)	
uppi	(I) tempo	(J) tempo	Differenza fra medie (I-J)	Errore std.	Sig.(a)	Limite inferiore	Limite superiore
00	1	2	-,172	,194	1,000	-,673	,329
		3	-,196	,198	,993	-,707	,314

	2	1	,172	,194	1,000	-,329	,673
		3	-,024	,146	1,000	-,401	,352
	3	1	,196	,198	,993	-,314	,707
		2	,024	,146	1,000	-,352	,401
ͻ0	1	2	-,284	,219	,624	-,849	,282
		3	-,147	,223	1,000	-,723	,429
	2	1	,284	,219	,624	-,282	,849
		3	,136	,164	1,000	-,288	,561
	3	1	,147	,223	1,000	-,429	,723
		2	-,136	,164	1,000	-,561	,288

ɜato sulle medie marginali stimate
Ͻorrezione per confronti multipli: Bonferroni.

Test multivariati

uppi		Valore	F	Ipotesi df	Gradi di libertà dell'errore	Sig.
ͻ0	Traccia di Pillai	,043	,500(a)	2,000	22,000	,613
	Lambda di Wilks	,957	,500(a)	2,000	22,000	,613
	Traccia di Hotelling	,045	,500(a)	2,000	22,000	,613
	Radice di Roy	,045	,500(a)	2,000	22,000	,613
ͻ0	Traccia di Pillai	,074	,880(a)	2,000	22,000	,429
	Lambda di Wilks	,926	,880(a)	2,000	22,000	,429
	Traccia di Hotelling	,080	,880(a)	2,000	22,000	,429
	Radice di Roy	,080	,880(a)	2,000	22,000	,429

scun F verifica gli effetti multivariati semplici di tempo all'interno di ogni combinazione di livelli degli effetti illustrati. Questi test sono ɩati sui confronti a coppie indipendenti e lineari tra le medie marginali stimate.
Ꞩtatistica esatta

odello lineare generalizzato

Note

ɩtput creato		19-JUN-2014 11:28:08
ɔmmenti		
ɔut	Dati	H:\• CARTELLE PERSONALI\Rausa Marialuisa\Articolo MIO\analisi statistiche\analisi luana\database completato maggio 2014.sav
	File di dati attivo	FileDati1
	Filtro	<nessuno>
	Peso	<nessuno>
	Distingui	<nessuno>
	N. di righe nel file dati di lavoro	31
ɜstione valori ɜancanti	Definizione di valore mancante	I valori mancanti definiti dall'utente vengono considerati mancanti.
	Casi utilizzati	Le statistiche sono basate su tutti i casi con dati validi di tutte le variabili del modello.

ntassi		GLM FSkmr_T1 FSkmr_T3 FSkmr_T4 BY gruppi<br>/WSFACTOR=tempo 3 Polynomial<br>/MEASURE=mr<br>/METHOD=SSTYPE(3)<br>/EMMEANS=TABLES(gruppi) COMPARE ADJ(BONFERRONI)<br>/EMMEANS=TABLES(tempo) COMPARE ADJ(BONFERRONI)<br>/EMMEANS=TABLES(gruppi*tempo) COMPARE(gruppi) ADJ(BONFERRONI)<br>/EMMEANS=TABLES(gruppi*tempo) COMPARE(tempo) ADJ(BONFERRONI)<br>/PRINT=DESCRIPTIVE<br>/CRITERIA=ALPHA(.05)<br>/WSDESIGN=tempo<br>/DESIGN=gruppi.
sorse	Tempo trascorso	0:00:00,03

```
ileDati1] H:\• CARTELLE PERSONALI\Rausa Marialuisa\Articolo MIO\analisi
atistiche\analisi luana\database completato maggio 2014.sav
```

Fattori entro soggetticc

ura: mr

npo	Variabile dipendente
	FSkmr_t1
	FSkmr_t3
	FSkmr_t4

Fattori tra soggetti

		N
uppi	1,00	15
	2,00	11

Statistiche descrittive

	gruppi	Media	Deviazione std.	N
;kmr_t1	1,00	2,3527	,86437	15
	2,00	2,9336	,92911	11
	Totale	2,5985	,92161	26
;kmr_t3	1,00	1,9273	,99106	15
	2,00	2,7791	,92390	11
	Totale	2,2877	1,03713	26
;kmr_t4	1,00	1,7187	,82411	15

2,00	2,6764	,61917	11
Totale	2,1238	,87550	26

Test multivariati(b)

fetto		Valore	F	Ipotesi df	Gradi di libertà dell'errore	Sig.
npo	Traccia di Pillai	,181	2,546(a)	2,000	23,000	,100
	Lambda di Wilks	,819	2,546(a)	2,000	23,000	,100
	Traccia di Hotelling	,221	2,546(a)	2,000	23,000	,100
	Radice di Roy	,221	2,546(a)	2,000	23,000	,100
npo * gruppi	Traccia di Pillai	,038	,456(a)	2,000	23,000	,640
	Lambda di Wilks	,962	,456(a)	2,000	23,000	,640
	Traccia di Hotelling	,040	,456(a)	2,000	23,000	,640
	Radice di Roy	,040	,456(a)	2,000	23,000	,640

Statistica esatta
)isegno: Intercept+gruppi
segno entro soggetti: tempo

Test di sfericità di Mauchly(b)

ura: mr

fetto entro soggetti	W di Mauchly	Approssimazione chi-quadrato	df	Sig.	Epsilon(a)		
					Greenhouse-Geisser	Huynh-Feldt	Limite inferiore
npo	,952	1,139	2	,566	,954	1,000	,500

ifica l'ipotesi nulla per la quale la matrice di covarianza dell'errore della variabile dipendente trasformata ortonormalizzata è
porzionale a una matrice identità.
E possibile utilizzarlo per regolare i gradi di libertà per i test di significatività mediati. I test corretti vengono visualizzati nella tabella dei
: sugli effetti entro soggetti.
)isegno: Intercept+gruppi
segno entro soggetti: tempo

Test degli effetti entro soggetti

ura: mr

rgente		Somma dei quadrati Tipo III	df	Media dei quadrati	F	Sig.
npo	Assumendo la sfericità	2,597	2	1,298	2,879	,066
	Greenhouse-Geisser	2,597	1,908	1,361	2,879	,069
	Huynh-Feldt	2,597	2,000	1,298	2,879	,066
	Limite inferiore	2,597	1,000	2,597	2,879	,103
npo * gruppi	Assumendo la sfericità	,479	2	,240	,531	,591
	Greenhouse-Geisser	,479	1,908	,251	,531	,583
	Huynh-Feldt	,479	2,000	,240	,531	,591
	Limite inferiore	,479	1,000	,479	,531	,473
rore(tempo)	Assumendo la sfericità	21,650	48	,451		

	Greenhouse-Geisser	21,650	45,788	,473		
	Huynh-Feldt	21,650	48,000	,451		
	Limite inferiore	21,650	24,000	,902		

Test dei contrasti entro soggetti

ura: mr

ɔrgente	tempo	Somma dei quadrati Tipo III	df	Media dei quadrati	F	Sig.
ηpo	Lineare	2,521	1	2,521	5,309	,030
	Quadratico	,076	1	,076	,178	,676
ηpo * gruppi	Lineare	,450	1	,450	,948	,340
	Quadratico	,029	1	,029	,067	,798
rore(tempo)	Lineare	11,395	24	,475		
	Quadratico	10,255	24	,427		

Test degli effetti fra soggetti

ura: mr
iabile trasformata: Media

ɔrgente	Somma dei quadrati Tipo III	df	Media dei quadrati	F	Sig.
tercetta	437,901	1	437,901	317,791	,000
uppi	12,088	1	12,088	8,772	,007
rore	33,071	24	1,378		

edie marginali attese

gruppi

Stime

ura: mr

			Intervallo di confidenza 95%	
uppi	Media	Errore std.	Limite inferiore	Limite superiore
)0	2,000	,175	1,638	2,361
)0	2,796	,204	2,375	3,218

Confronti a coppie

ura: mr

gruppi	(J) gruppi	Differenza fra medie (I-J)	Errore std.	Sig.(a)	Intervallo di confidenza per la differenza al 95%(a)

					Limite inferiore	Limite superiore
)0	2,00	-,797(*)	,269	,007	-1,352	-,242
)0	1,00	,797(*)	,269	,007	,242	1,352

ɜato sulle medie marginali stimate
a differenza fra medie è significativa al livello ,05
Ↄorrezione per confronti multipli: Bonferroni.

Test univariati

ura: mr

	Somma dei quadrati	df	Media dei quadrati	F	Sig.
ɔntrasto	4,029	1	4,029	8,772	,007
rore	11,024	24	,459		

scun F verifica gli effetti semplici di gruppi all'interno di ogni combinazione di livelli degli altri effetti illustrati. Questi test sono basati confronti a coppie indipendenti e lineari tra le medie marginali stimate.

tempo

Stime

ura: mr

			Intervallo di confidenza 95%	
mpo	Media	Errore std.	Limite inferiore	Limite superiore
	2,643	,177	2,278	3,009
	2,353	,191	1,958	2,748
	2,198	,148	1,892	2,503

Confronti a coppie

ura: mr

					Intervallo di confidenza per la differenza al 95%(a)	
tempo	(J) tempo	Differenza fra medie (I-J)	Errore std.	Sig.(a)	Limite inferiore	Limite superiore
	2	,290	,203	,498	-,232	,812
	3	,446	,193	,091	-,052	,943
	1	-,290	,203	,498	-,812	,232
	3	,156	,167	1,000	-,275	,587
	1	-,446	,193	,091	-,943	,052
	2	-,156	,167	1,000	-,587	,275

ɜato sulle medie marginali stimate
Ↄorrezione per confronti multipli: Bonferroni.

Test multivariati

	Valore	F	Ipotesi df	Gradi di libertà dell'errore	Sig.
accia di Pillai	,181	2,546(a)	2,000	23,000	,100
ımbda di Wilks	,819	2,546(a)	2,000	23,000	,100
accia di Hotelling	,221	2,546(a)	2,000	23,000	,100
adice di Roy	,221	2,546(a)	2,000	23,000	,100

scun F verifica l'effetto multivariato di tempo. Questi test sono basati sui confronti a coppie indipendenti e lineari tra le medie rginali stimate.
Statistica esatta

gruppi * tempo

Stime

ura: mr

uppi	tempo	Media	Errore std.	Intervallo di confidenza 95% Limite inferiore	Limite superiore
00	1	2,353	,230	1,877	2,828
	2	1,927	,249	1,414	2,441
	3	1,719	,193	1,321	2,116
00	1	2,934	,269	2,379	3,489
	2	2,779	,291	2,179	3,379
	3	2,676	,225	2,212	3,140

Confronti a coppie

ura: mr

mpo	(I) gruppi	(J) gruppi	Differenza fra medie (I-J)	Errore std.	Sig.(a)	Intervallo di confidenza per la differenza al 95%(a) Limite inferiore	Limite superiore
	1,00	2,00	-,581	,354	,114	-1,312	,150
	2,00	1,00	,581	,354	,114	-,150	1,312
	1,00	2,00	-,852(*)	,383	,036	-1,641	-,062
	2,00	1,00	,852(*)	,383	,036	,062	1,641
	1,00	2,00	-,958(*)	,296	,004	-1,569	-,347
	2,00	1,00	,958(*)	,296	,004	,347	1,569

sato sulle medie marginali stimate
a differenza fra medie è significativa al livello ,05
Correzione per confronti multipli: Bonferroni.

Test univariati

ura: mr

mpo		Somma dei quadrati	df	Media dei quadrati	F	Sig.
	Contrasto	2,142	1	2,142	2,693	,114

Errore	19,092	24	,796		
Contrasto	4,604	1	4,604	4,958	,036
Errore	22,287	24	,929		
Contrasto	5,821	1	5,821	10,470	,004
Errore	13,342	24	,556		

erifica l'effetto di gruppi. Questo test è basato sui confronti a coppie indipendenti e lineari tra le medie marginali stimate.

gruppi * tempo

Stime

ura: mr

uppi	tempo	Media	Errore std.	Intervallo di confidenza 95%	
				Limite inferiore	Limite superiore
00	1	2,353	,230	1,877	2,828
	2	1,927	,249	1,414	2,441
	3	1,719	,193	1,321	2,116
00	1	2,934	,269	2,379	3,489
	2	2,779	,291	2,179	3,379
	3	2,676	,225	2,212	3,140

Confronti a coppie

ura: mr

uppi	(I) tempo	(J) tempo	Differenza fra medie (I-J)	Errore std.	Sig.(a)	Intervallo di confidenza per la differenza al 95%(a)	
						Limite inferiore	Limite superiore
00	1	2	,425	,264	,360	-,254	1,105
		3	,634	,252	,056	-,014	1,282
	2	1	-,425	,264	,360	-1,105	,254
		3	,209	,218	1,000	-,352	,769
	3	1	-,634	,252	,056	-1,282	,014
		2	-,209	,218	1,000	-,769	,352
00	1	2	,155	,308	1,000	-,639	,948
		3	,257	,294	1,000	-,499	1,013
	2	1	-,155	,308	1,000	-,948	,639
		3	,103	,254	1,000	-,552	,757
	3	1	-,257	,294	1,000	-1,013	,499
		2	-,103	,254	1,000	-,757	,552

sato sulle medie marginali stimate
Correzione per confronti multipli: Bonferroni.

Test multivariati

uppi		Valore	F	Ipotesi df	Gradi di libertà dell'errore	Sig.
)0	Traccia di Pillai	,209	3,043(a)	2,000	23,000	,067
	Lambda di Wilks	,791	3,043(a)	2,000	23,000	,067
	Traccia di Hotelling	,265	3,043(a)	2,000	23,000	,067
	Radice di Roy	,265	3,043(a)	2,000	23,000	,067
)0	Traccia di Pillai	,031	,371(a)	2,000	23,000	,694
	Lambda di Wilks	,969	,371(a)	2,000	23,000	,694
	Traccia di Hotelling	,032	,371(a)	2,000	23,000	,694
	Radice di Roy	,032	,371(a)	2,000	23,000	,694

scun F verifica gli effetti multivariati semplici di tempo all'interno di ogni combinazione di livelli degli effetti illustrati. Questi test sono ;ati sui confronti a coppie indipendenti e lineari tra le medie marginali stimate.
;tatistica esatta
